# Deciphering Main Climate and Edaphic Components Driving Oat Adaptation to Mediterranean Environments

**DOI:** 10.3389/fpls.2021.780562

**Published:** 2021-11-26

**Authors:** Francisco J. Canales, Gracia Montilla-Bascón, Luis M. Gallego-Sánchez, Fernando Flores, Nicolas Rispail, Elena Prats

**Affiliations:** ^1^CSIC, Institute of Sustainable Agriculture, Córdoba, Spain; ^2^E.T.S.I. El Carmen, University of Huelva, Huelva, Spain

**Keywords:** adaptation, Mediterranean climate, genotype × environment interaction, variation partitioning, Oat (*A. sativa* L.), landrace

## Abstract

Oat, *Avena sativa*, is an important crop traditionally grown in cool-temperate regions. However, its cultivated area in the Mediterranean rim steadily increased during the last 20 years due to its good adaptation to a wide range of soils. Nevertheless, under Mediterranean cultivation conditions, oats have to face high temperatures and drought episodes that reduce its yield as compared with northern regions. Therefore, oat crop needs to be improved for adaptation to Mediterranean environments. In this work, we investigated the influence of climatic and edaphic variables on a collection of 709 Mediterranean landraces and cultivars growing under Mediterranean conditions. We performed genotype–environment interaction analysis using heritability-adjusted genotype plus genotype–environment biplot analyses to determine the best performing accessions. Further, their local adaptation to different environmental variables and the partial contribution of climate and edaphic factors to the different agronomic traits was determined through canonical correspondence, redundancy analysis, and variation partitioning. Here, we show that northern bred elite cultivars were not among the best performing accessions in Mediterranean environments, with several landraces outyielding these. While all the best performing cultivars had early flowering, this was not the case for all the best performing landraces, which showed different patterns of adaption to Mediterranean agroclimatic conditions. Thus, higher yielding landraces showed adaptation to moderate to low levels of rain during pre- and post-flowering periods and moderate to high temperature and radiation during post-flowering period. This analysis also highlights landraces adapted to more extreme environmental conditions. The study allowed the selection of oat genotypes adapted to different climate and edaphic factors, reducing undesired effect of environmental variables on agronomic traits and highlights the usefulness of variation partitioning for selecting genotypes adapted to specific climate and edaphic conditions.

## Introduction

Oat is an important cereal crop ranking seventh in the world and fourth in Europe regarding cultivated area, with more than 10 million Ha worldwide (FAO, 2017). It has been used as food and fodder since ancient times. As food, oat is being increasingly appreciated as part of different products including porridge, breakfast cereals, breads, muffins, cookies, snacks, meat extenders, beverages, and baby foods for its benefits on human health (Londono et al., 2015; Gilissen et al., 2016; Martinez-Villaluenga and Peñas, 2017). Regarding livestock, oat makes good hay and silage and it is used as feed, fodder, and straw. *Avena sativa*, the main cultivated oat species includes white oats, preferred for milling and used for human food and fodder, and the red oats (formerly known as *A. byzantina* K. Koch) preferred for hay (Stevens et al., 2004). Although oat center of origin is in the fertile crescent, it is argued that the cultivated hexaploid species, evolved during the migration of its ancestors northward, where it was established as a primary crop (Thomas, 1995). Thus, *A. sativa* is mostly grown as spring-sown crop in cool-temperate regions, under moist and cool conditions.

Surprisingly, the oat-cultivated area in the Mediterranean rim has steadily increased during the last 20 years reaching similar harvested area to that of the Northern European countries (FAO, 2017). This is in part due to its good adaptation to a wide range of soil types and because on marginal soils oat can outperform other small-grain cereals (Stevens et al., 2004). However, oat water requirements are among the highest of small grain cereals due to its high transpiration rate (Ehlers, 1989). This, together with the fact that most of the current cultivars used as winter crop in the Mediterranean rim are spring cultivars bred in Northern countries might explain the poor oat adaptation observed in this area. Under Mediterranean cultivation conditions, oat has to face high temperatures and drought that cause grain abortion (Sánchez-Martín et al., 2017). All the above could explain the almost three-fold lower yield observed in Mediterranean rim when compared with Northern regions.

In contrast to the low adaptation observed in many oat cultivars growing under Mediterranean conditions, this area is rich in locally well-adapted oat landraces. Plant landraces are regional and locally adapted ecotypes that have adapted to their natural environment over time. Thus, landrace populations have evolved in response to specific environmental conditions following a pattern of local adaptation (Williams, 1966). They are recognized to present a tangible crop genetic resource of actual or potential economic benefit for human kind and provide a large gene pool for future genetic breeding programs and food security (Ceccarelli, 1994; Chalak et al., 2015). Landrace performance may exceed those of the varieties derived from them in terms of adaptation to changes in environmental conditions and agricultural system (Kneupffer et al., 2003). However, during the last century, landraces have often been replaced by modern cultivars reducing the diversity of several crops including wheat, barley, and maize (revised by Newton et al., 2010). The diversity loss is becoming even more important nowadays as we face the need to adapt crops to climate change (Frison et al., 2011; Ceccarelli, 2012; Dwivedi et al., 2016). After the adoption of modern cultivars, landraces moved into unfavorable and stress-prone environments (Li et al., 2012). Thus, their assessment could reveal key aspects about adaptation to low-input environments, such as the Mediterranean oat crop system. While under optimal conditions modern cultivars may outyield landraces, under low input systems, landraces may show higher or similar yield than modern cultivars, as confirmed in barley (Yahiaoui et al., 2014) and oat (Rispail et al., 2018).

Although landraces constitute one of the most valuable sources of genetic diversity, their potential value for environmental adaptation has not been fully appreciated or appropriately documented. Further studies are thus necessary regarding the assessment of landraces of particular crops for their adaptation to environment. From the phenotyping point of view, there is a need to determine their genotype × environment interaction (GEI) since it attenuates the association between phenotype and genotype, reducing genetic progress in plant breeding programs. Gaining knowledge on how GEI affects the performance of genotypes may aid selecting the best genotypes and defining their optimum environments to maximize yield. This can be implemented through the additive main effects and multiplicative interaction (AMMI) models and GGE Biplots analyses (Gauch et al., 2008). Both, AMMI and GGE biplots are based on fixed-effects models with (additive) main effects for genotypes and environments and multiplicative effects for the interaction, all being fixed (Yang et al., 2009). However, it is frequently assumed that environments are random because the environment included in the multienvironmental trials is only a sample of a large environment. The use of mixed models using restricted maximum likelihood methodology (REML) algorithm, which considers the environment effects as random, and the Best Linear Unbiased Prediction (BLUP), which predicts the outcome of random variables, solve this problem (Parsad et al., 2009). Thus, GGE analyses have been previously proven useful to characterize disease resistance and to select breeding material for yield stability and other agronomic traits in field trials of oat (Sánchez-Martín et al., 2014, 2017; Rispail et al., 2018) and other species (Villegas-Fernandez et al., 2009; Fernandez-Aparicio et al., 2012; Flores et al., 2012, 2013; Rubiales et al., 2012, 2014).

While on a large spatial scale, climate is one of the main sources of environmental variation (Santamaria et al., 2003), on small scale, different edaphic or biotic conditions have a key role on environmental variation and local plant adaptation (Bell et al., 2000; van der Putten et al., 2004). Particular climatic factors such as precipitation and temperature may determine the composition of the vegetation in a particular environment (Box, 1996). On the other hand, soil abiotic variables, such as nutrient content, structure, and composition, but also biotic soil properties may act as selective agents influencing plant performance and adaptation (Wardle, 2002; Macel et al., 2007). The influence of these different variables on local adaptation can be separated by regression-based methods between others. However, the use of partial regression is questionable for intercorrelated variables, which is common in nature because of the interaction among explanatory variables (Zar, 1984). Canonical ordination and more specifically variation partitioning allow the partition of the observed variation among different variables, such as climate and edaphic variables, and is commonly used to explore the relationships between biological phenomena and environmental influences (Legendre, 2008).

To assess the contribution of environmental and genetic variation and their interaction to phenotypic variation and agronomic performance in oat, we carried out multiyear and multisite trials involving a collection of 709 white and red *A. sativa* accessions, including mainly landraces and 65 cultivars for comparison. The main aim is to provide the theoretical basis to test oat performance in Mediterranean environments and to explain the local adaptation of the best performing landraces according to the influence exerted on their performance by different climate and edaphic factors.

## Materials and Methods

### Plant Material and Experimental Design

**FIGURE 1:**
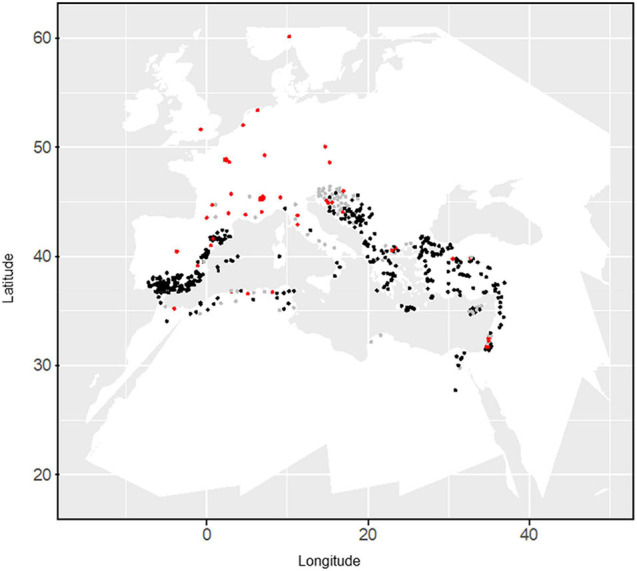
Distribution of the oat collection. Landraces and cultivars are indicated by black and red dots, respectively. Gray dots indicate uncertain type of material. Localization of cultivars was that of the developing company.

An oat collection consisting of 709 white and red oats, with both landraces from the Mediterranean area and cultivars were used (Figure 1). Seeds were provided by “Centro de Recursos Fitogenéticos” (INIA, Madrid) and United States Department of Agriculture (USDA, Washington) germplasm banks. Cultivars included within the collection, were provided by different institutions and reported in Sánchez-Martín et al. (2014). Several of them were bred in Northern countries (Figure 1) and were included for comparison since they are widely cultivated in the Mediterranean area. An interactive map with the 709 oat accessions was created with Google Maps (Google-LTD 2019) (Canales et al., 2021) and can be accessed at https://www.google.com/maps/d/viewer?mid=1t-O7OUoUPJ_qY5qGQsq66O5zg7k. It contains information of location and other passport data. Further details on genetic relationships and population structure of this oat collection can be found in Montilla-Bascón et al. (2013) and Canales et al. (2021).

The oat collection was evaluated for main agronomic traits in three different environments in Spain. Trials were performed in two contrasting locations, Santaella, with 238 m altitude and light clay eutric gleysol during the growing season between 2017 and 2018 (SA18) and Cordoba with 90 m altitude and light clay calcic cambisol during growing seasons between 2016–2017 (CO17) and 2017–2018 (CO18). At each location–year, an alpha lattice square design with three replicates was used and the cultivar Patones was used as check. The randomization of genotypes was done with SPSS v. 25 software (IBM Corp., Armonk, NY, United States). Each replicate consisted of 27 × 27 1-m-long rows containing the 709 genotypes plus additional checks included in each row and column until completing the lattice square. Rows were separated from each other by 30 cm, at a sowing density of 260 seeds m^–2^. Replications were bordered by cv Flega. Sowings took place in December according to local practices. No irrigation was performed in the trials. Hand weeding was carried out when required, and no herbicides or fertilizers were applied. Trials were hand-harvested to avoid seed mixture.

### Agronomic Trait Assessment

At maturity stage, total above-ground dry matter was determined following field-drying of the plant material for at least 1 week. All grain was oven-dried at 70°C. Yield is presented on an oven-dry basis of seeds weight (kg/ha). Biomass data are based on the above-ground plant weight (kg/ha). Earliness was estimated as days to heading by counting the number of days from sowing to heading. Height was measured with a ruler measuring from the ground to the end of the spike. When grain abortion was observed, severity was assessed as a visual estimation of the percentage of whole grain aborted per plant before harvest.

### Environmental Variables

To evaluate the influence of environmental factors on the agronomic traits, climate and edaphic variables from the different tested environments were recorded. Six climate variables (average temperature, rain, and radiation during pre- and post-flowering period) were gathered for each location from the AEMET^1^, and Junta de Andalucía^2^ databases. Soil measurements were performed in three soil samples per replicate. Each soil sample consisted of a pool of five subsamples randomly distributed over the plot at the first 40-cm depth. Samples collected from each environment were analyzed to determine soil parameters related with both nutrient content and chemical characteristics (organic matter, pH, cationic exchange, nitrogen, potassium, and phosphorus) by the Agro-food laboratory service of the regional government (AGAPA, Junta de Andalucía, Spain).

### Statistical Analysis

#### Variance Analyses

All analyses of variance (ANOVA) were performed using SAS 9.3 (SAS Institute Inc., Cary, NC, United States). ANOVA for each environment was performed to generate LSMEANS using PROC MIXED with genotype as a fixed effect and blocks (replicate) as a random effect. Combined ANOVA was performed for all traits using PROC MIXED with genotype as the fixed effect, and environment, rep (environment), block (environment*rep), and genotype by environment interaction as random effects using a REML algorithm. Best linear unbiased predictor (BLUP) for genotype by environment interaction was computed. HA-GGE biplot analysis was carried out on the genotype by environment table of BLUPs. Pearson correlations were calculated to detect statistical correlations between traits.

#### HA–GGE Biplots

The HA–GGE biplot method (Yan and Holland, 2010) was chosen here since it takes into consideration any heterogeneity among environments by giving weights to the test environments proportional to their root square heritability (Sánchez-Martín et al., 2014). Therefore, it is most appropriate for visual evaluation of the test environments and genotypes. Analyses were made with the SAS 9.3 (SAS Institute Inc., Cary, NC, United States) program developed by Burgueño et al. (2003) to graph GGE biplots. The prediction of the outcome of random variables was done by the Best Linear Unbiased Prediction (BLUP), as originally suggested by Henderson (1975). The target environment axis is represented by a corresponding red straight line drawn through the biplot origin, and the average environment (TEAa) defines the mean ordinates of all environments in the biplot. Genotypes located on the polygon vertices reveal the best or the poorest for a particular environment.

#### Canonical Correspondence and Redundancy Analysis

Canonical correspondence analysis (CCA) is a multivariate method that allows elucidation of the relationship between a factor (biological individual, marker, or trait) and the environment extracting synthetic environmental gradients from the datasets. These gradients are the basis to visualize the factor–environment relationship through ordination diagrams offering additional information about the particular environmental variables that influence the factor behavior. CCA was used to establish the effect of the environmental estimates on the different agronomic traits and accession performances. Analyses were made with PAST software (Hammer et al., 2001). For CCA analysis of heading date, the climatic variables considered were those recorded until heading.

The association of the different accessions with particular environmental variables was further analyzed by constrained ordination redundancy analysis (RDA) using “vegan” library (Dixon, 2003; Oksanen et al., 2020) of the statistical language R v3.6.0 (R Development Core Team, 2008) and according to Borcard et al. (2017). A Monte Carlo test (999 permutations) based on the RDA was used to assess the effects of each variable. This technique is widely used to test whether the variation in one set of (independent) variables explains the variation in another set of (dependent) variables (Leamy et al., 2016). The different agronomic traits were considered as the dependent variables and the set of 12 agroclimatic variables comprised the independent set. Following the same procedure performed by Lasky et al. (2015), an independent assessment of the environmental impact on the different agronomic traits among accessions was performed by comparing one complete and two partial RDA models for each of the climatic and soil variables. Thus, for climatic variables, the models included, in addition to the *Q* values matrix, either the total set of climatic variables, or a partial set comprising variables recorded at pre or post-flowering period. Similarly, the models tested for edaphic variables included either all variables or a partial set comprising either the assimilable nutrient content variables (potassium, nitrogen, and phosphorus) or the chemical characteristics (cationic exchange capacity, pH, and organic matter). By comparing the six models, the common and independent contributions of climatic and soil variables on the distribution of the different accessions for each agronomic factor could be estimated. RDA was performed using a permutational ANOVA-like test on redundancy-analysis fitted data (function anova.cca) to test the significance of the effect of climatic and soil variables on the distribution of the different accessions for each agronomic factor. In addition, we implemented a variation partitioning analysis to assess the relative importance of the influence of climate and soil influence for each agronomic factor. Partitioning variation analyses were carried out with the “vegan” package in R according to the procedure developed by Borcard et al. (2017). All variables were represented in the figures in order to infer their relationships, but only non-collinear variables were considered for further analysis (Borcard et al., 2017).

## Results

### Differences Among Environments Tested Regarding Climate and Edaphic Factors

**FIGURE 2:**
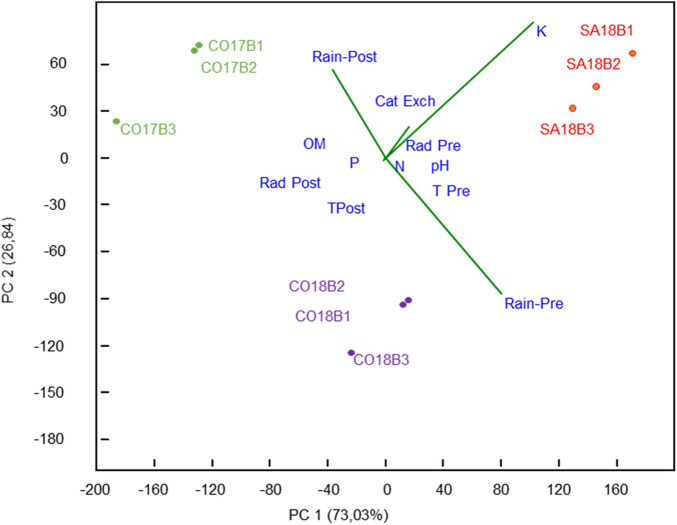
Climatic/edaphic environmental differences in the multitrial sites. Principal component analysis showed differences among the environments tested (CO17, CO18, and SA18) regarding climate and edaphic factors highlighting the variables with highest weight in the distribution. Climate variables include: *T* (average temperature), Rad (radiation), and rain during pre- (Rain-Pre) or post-flowering period (Rain-Post). Edaphic variables include the content in OM (organic matter), K (assimilable potassium), N (nitrogen), P (phosphorous), Cat Exch (Cationic exchange capacity), and pH. Replicated trials from the same locality (B1, B2, and B3) are represented with same color.

Principal component analysis showed differences among the three environments assessed (CO17, CO18, and SA18) regarding climate and edaphic factors highlighting the variables with the highest weight in the distribution. Figure 2 showed that the first two PCs explain a 99.87% of the observed variability (73.03% in PC 1 and 26.84% in PC 2). PC 1 and PC 2 split the three different environments tested, where rain pre and post-flowering and potassium content (longer vectors in the PCA) had a higher weight in the distribution (Figure 2). Environment CO17 showed high levels of rain post-flowering and low levels of rain preflowering, with low potassium content and cation exchange, whereas environment CO18 and SA18 were characterized by similarly low levels of rain post-flowering and opposed content of potassium and cation exchange.

### Genotype–Environment Interactions of Oat Cultivars and Landraces Growing Under Mediterranean Conditions

**FIGURE 3:**
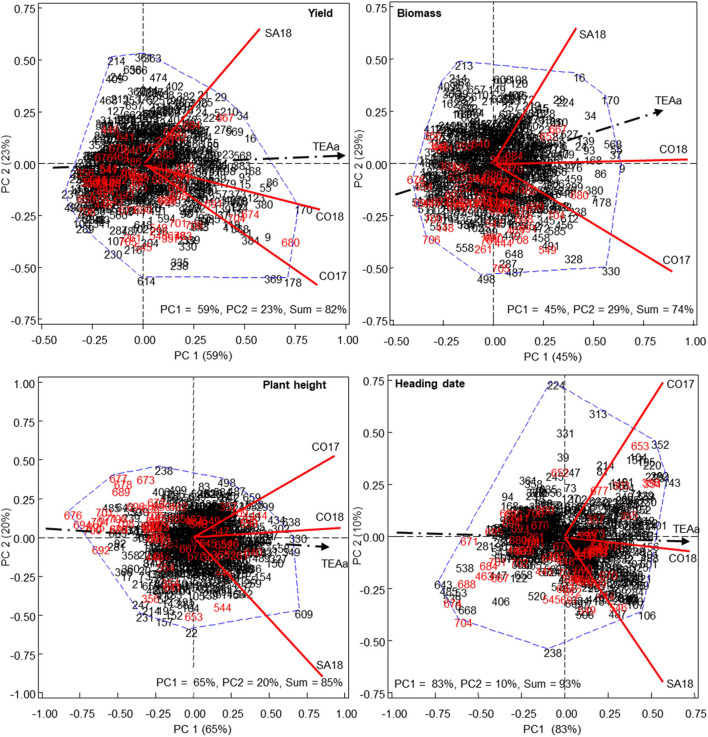
HA-GGE biplot of different agronomic traits. HA-GGE biplot based on the grain yield (kg/ha), biomass (Tones/Ha), H Index (HI), plant height and heading date of 709 Mediterranean oat landraces and varieties grown at three different environments, from 2017 (17) to 2018 (18). (Cordoba, CO; Santaella, SA). Numbers in black and red indicate landraces and cultivars, respectively.

Combined analysis of variance according to REML methods showed genotype x environment interaction for all assessed traits (*P* < 0.001 in all cases). Thus, further analysis were performed combining ANOVA and principal component (PC) approaches to graphically display G and GE interactions and to identify candidate genotypes with desirable and consistent performance across environments for each trait. As observed in Figure 3, the first two PCs captured a high percentage of the total variability, ranging from 74% for biomass to 93% for heading date.

For the different agronomic traits, we observed a different distribution of accessions along the TEAa axis and each particular tested environment (Figure 3). Positive and negative projections onto the TEAa indicated high or low levels of the considered trait whereas the angle formed between the accession and the vector indicated repeatability. For yield and biomass, most cultivars (depicted in red) grouped on the left side of the TEAa showing negative or short positive projections over this axis. This indicated the low performance of most of these cultivars in the average environment. Interestingly, some of the cultivars that showed higher yield such as 667, 680, and 704 (namely Precoce Maroc, Cassandra and Rapidena) were breed in Morocco, Greece, and Spain, respectively, whereas most of the cultivars with negative projections had been bred in Northern countries. Accession 674, which also showed high yield is cultivar Alcudia, bred in France, but highly resistant to crown rust which might be related with the high yield in Mediterranean environments where rust disease cause significant yield losses. The results obtained for biomass followed a similar trend. As for yield, cultivars 667, 680, and 704 were among those with higher biomass (Figure 3). In this case, the biplot also highlighted the Mediterranean cultivar 652 (Bozkir), which was bred in Turkey.

Interestingly, many landraces outyielded the above-mentioned best cultivars. The highest yielding landraces with the highest projections on TEAa axis were 9, 55, 86, 168, 170, and 178 collected in Spain, and the Greek landraces 369 and 380. Accessions 178 and 369 showed high angle with TEAa, indicating lower repeatability for the average environment but the best results in the particular environmental conditions of CO17, characterized by lower preflowering rain but higher post-flowering rain compared with SA18 (Figure 2). By contrast, the Spanish accession 29 showed good yield regarding the average environment but higher projection and lower angle with respect to SA18. This indicated that these landraces were adapted to particular characteristics of this environment. Regarding biomass, we also detected several landraces that performed better than the best cultivars. Most of them such as 9, 16, 34, 55, 86, 170, or 178 were also higher grain yielding (Figure 3). Accordingly, a high and significant correlation was observed between yield and biomass with *R* = 0.64 (*P* < 0.001).

Plant height and heading date biplots showed a wider distribution of cultivars on both side of the TEAa. Interestingly, the accessions with the lowest height corresponded to cultivars including 676, 694, 695, 700, or 704 (namely Selma, Karmela, Pallini, Kazmina, and Rapidena, respectively), whereas the accessions with the highest height were landraces including 330, 349, and 638 (Figure 3). A significant correlation was observed between height and biomass (*R* = 0.61, *P* < 0.001), with the highest biomass corresponding overall to the tallest accessions. By contrast the early flowering group was composed of both cultivars and landraces including the cultivars 688, 671, 674, and 704 (namely Flega, Acebeda, Alcudia, and Rapidena, respectively) and the landraces 485, 538, 539, and 643 originated from Libya, Cyprus, and Turkey, respectively. Interestingly, the best performing accessions were those with the earliest heading date, with both yield and biomass showing a negative and significant correlation with heading date (*R* = −0.64, *P* < 0.001 and *R* = −0.55, *P* < 0.001 for yield and biomass, respectively).

The performance of white and red oat accessions (subspecies *sativa* and *byzantina*, respectively) was also compared for each agronomic trait. No subspecies-specific grouping was evidenced for any of the agronomic traits at individual scale. Instead, GGE-biplots showed that the best-performing accessions for each agronomic trait are members of the two subspecies. For instance, two of the above mentioned high yielding accessions for the average environment (380 and 680) are red oats whereas the high yielding accessions 55, 86, 170, and 178 belonged to the white oat group. However, taken together, red oats showed on average slightly higher performance than white oats. Thus, red oats were significantly higher yielding (approximately 18%, *P* < 0.001) than white oats. Red oats also flowered almost 1 week earlier on average than white oats (*P* < 0.001), which might contribute to their higher yield. In addition, red oats produced slightly higher biomass (c.a. 6.5%, *P* < 0.001) which might be related to their slightly higher height (around 2 cm higher, *P* < 0.001).

### Variance Partitioning of Climate and Edaphic Factors on Agronomic Traits

**FIGURE 4:**
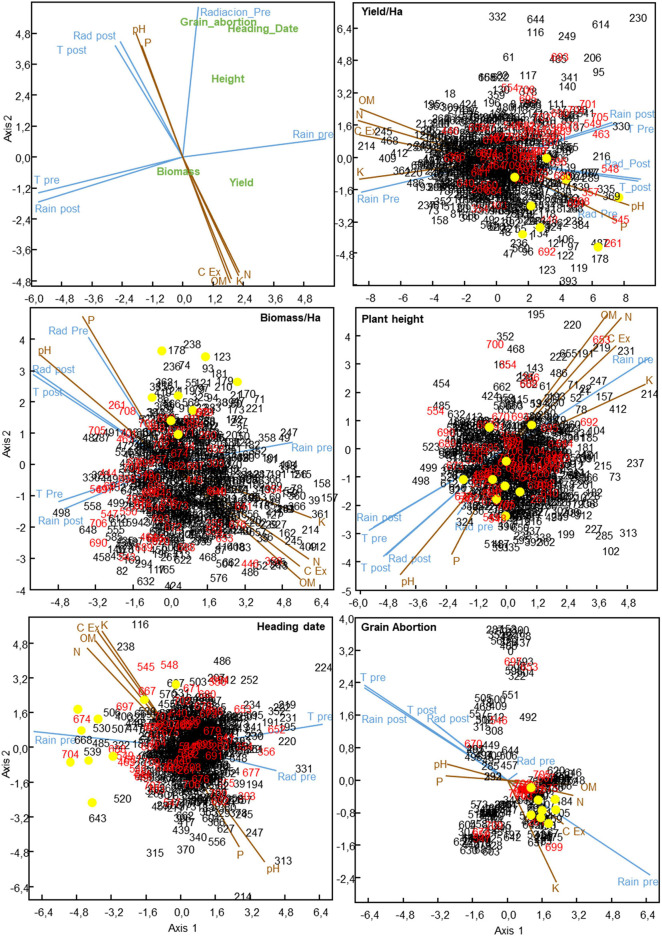
Canonical correspondence analysis of 709 Mediterranean oat landraces and cultivars and the influence of environmental factors in several agronomic traits. Numbers in black and red indicate landraces and cultivars, respectively. Yellow dots represent the best performing landraces on the average environment in terms of grain yield and biomass, and the tallest, earliest and those with the lowest grain abortion accessions, respectively, according to biplot analysis.

Redundancy analysis coupled with variance partitioning analysis was carried out to assess the significance of each environmental variable and the relative contribution of both climate and soil characteristics to the different agronomic traits (Tables 1, 2; left up panel of Figure 4). Regarding yield, we observed that the combination of climate and edaphic variables significantly explained almost 52% of the observed variation. Climatic factors explained the largest fraction of yield variation reaching almost 40% with a pure effect of 31% (Table 1). According to the model, the most important variables significantly determining yield were the amount of rain during preflowering period, the radiation post-flowering, and the soil organic matter content (Table 2). The partial models (i.e., pre- or post-flowering climate variables and chemical or nutrient soil variables) highlighted some additional significant factors accounting for the observed variation for all traits. For yield, these partial models revealed that the level of radiation preflowering and rain post-flowering together with the soil nitrogen content also significantly contributed to yield (Table 2). Interestingly, the variation partitioning observed for heading date was very similar to that of yield with the combination of climate and soil variables significantly explaining 53% of the observed variation while climate factors accounted for around 40% (Table 1). Nevertheless, the major determinant variables of flowering time were temperature together with the level of rain preflowering and the nitrogen content (Table 2).

**TABLE 1:** Partitioning analysis of different agronomic traits according to the influence of various climate and edaphic factors.

		Partitioning
Yield	Climate + interaction	39.41%**
	Soil + interaction	21.27%*
	All	51.93%**
	Climate	30.66%**
	Soil	12.52% ns
	Interaction	8.75%
Biomass	Climate + interaction	19.14%**
	Soil + interaction	18.48%*
	All	34.67%**
	Climate	16.19% ns
	Soil	15.53% ns
	Interaction	2.95%
Plant height	Climate + interaction	19.86%*
	Soil + interaction	18.09%*
	All	33.62%**
	Climate	15.53%*
	Soil	13.76% ns
	Interaction	4.33%
Heading date	Climate + interaction	40%***
	Soil + interaction	18.5%*
	All	53.18%**
	Climate	34.18***
	Soil	12.18% ns
	Interaction	5.32%
Grain abortion	Climate + interaction	Ns
	Soil + interaction	ns
	All	ns
	Climate	ns
	Soil	ns
	Interaction	

*, **, *** indicate the significant factors highlighted by the model at P < 0.05, 0.1, and 0.001, respectively. The significance of the model is also indicated. Ns, non-significant differences.

**TABLE 2:** Redundancy analysis of different agronomic traits according to the influence of various climate and edaphic factors.

	Climate					Soil		
		All	Pre	Post		All	Nutrient	Chem
Yield	Rain Pre	*	**		N		**	
	Rad pre		*		P			
	Aver Tem Pre				K			
	Rain Post			*	OM	*		*
	Rad post	*		**	C Ex			
	Aver Tem Post				pH			
	Model	*P* < 0.001	*P* < 0.001	*P* = 0,003	Model	*P* = 0,017	*P* = 0,013	*P* = 0,017
Biomass	Rain Pre				N			
	Rad pre		*		P	**	**	
	Aver Tem Pre				K		**	
	Rain Post	*		**	OM			*
	Rad post	**		*	C Ex	**		*
	Aver Tem Post				pH			
	Model	*P* = 0.007	*P* = 0.005	*P* = 0.008	Model	*P* = 0.002	*P* < 0.001	*P* = 0.013
Plant height	Rain Pre	*	*		N		*	
	Rad pre				P			
	Aver Tem Pre	*			K	*	*	
	Rain Post				OM	*		
	Rad post				C Ex			
	Aver Tem Post				pH			
	Model	*P* = 0.003	*P* = 0.023	ns	Model	*P* = 0.005	*P* = 0.001	ns
Heading date	Rain pre	**			N	*	*	
	Rad pre				P			
	Aver Tem Pre				K			
					OM			*
					C Ex			
					pH			
	Model	*P* = 0.003			Model	*P* = 0.018	*P* = 0.023	*P* = 0.013
Grain abortion	Rain Pre	**	**		N			
	Rad pre				P			
	Aver Tem Pre				K			
	Rain Post			*	OM			
	Rad post				C Ex			
	Aver Tem Post			*	pH			
	Model	*P* = 0.001	*P* = 0.003	*P* = 0.003	Model	ns	ns	ns

*, **, indicate the significant factors highlighted by the model at P < 0.05, 0.1, respectively. The significance of the model is also indicated. Ns, non-significant differences.

Regarding biomass, the combination of climate and soil variables explained 34.6% of the variation observed. As for yield, climate significantly explained the largest fraction of the variation (around 20%). Height showed a partitioning similar to that of biomass although the most determinant variables for each trait differed (Table 1). Thus, the most important variables significantly impacting biomass were rain and radiation post-flowering, and the phosphorus content or cationic exchange capacity, while height was mainly determined by preflowering climate variables, soil potassium, and organic matter content (Table 2). Interestingly, the variation partitioning for grain abortion using the complete models was not significant, probably due to the low influence of soil variables for this trait (Table 1). However, the models taking into account climate variables highlighted the levels of rain during preflowering together with the temperature and rain post-flowering as crucial determinants for grain abortion (Table 2).

The left up panel of Figure 4 depicts the influence of climate and edaphic variables on the agronomic traits. The projection of each agronomic trait onto the variable vectors indicates the positive or negative effect exerted by the variable on that particular trait. We observed that climate showed slightly longer vectors than edaphic variables, confirming the higher influence of climate variables on oat agronomic traits observed in the partition analysis. This representation allows the graphical identification of collinearity among variables. For instance, cation exchange capacity and organic matter together with assimilable potassium and nitrogen showed a very small angle among them indicating their similar effect, opposite to that of pH and phosphorous content, on the different agronomic traits. Several trends supporting the results of the partitioning analysis can be extracted from the canonical correspondence analysis for the different agronomic traits. Thus, high yield was overall favored by high amount of rain during pre-flowering period together with low temperatures/radiation during the post-flowering period. As expected, grain abortion was mainly favored by the succession of a preflowering period with moderate amount of rain followed by a post-flowering period with low rain and high temperature. Heading date and height were mainly favored by high rain and radiation levels during preflowering period (Figure 4).

### Local Adaptation of Oat Cultivars and Landraces to Mediterranean Climate and Edaphic Factors

The remaining panels of Figure 4 show the climate and edaphic factors that mostly explained the observed behavior of the oat accessions. We observed that the vectors representing the climate and edaphic variables grouped similarly with that observed in left-up panel. Thus, temperature and radiation post-flowering showed collinearity, and also rain, post-flowering, and temperature during pre-flowering, indicating that these variables are correlated between them and exert an opposite effect to that of rain levels during the preflowering stage. From the edaphic point of view, cation exchange capacity showed collinearity with organic matter, assimilable potassium, and nitrogen content while their effects were mostly opposite to that of phosphorous and pH.

In order to identify the most relevant climate and edaphic variables driving local adaptation of the best performing oat accessions detected by GGE analysis, these were first highlighted as yellow dots in the plots (Figure 3). Interestingly, we observed that the best performing landraces for each agronomic trait were grouped in particular regions of different plots. Thus, the best yielding landraces (with higher projections on TEAa) such as, 9, 55, 86, 170, 178, 369, and 680, were grouped in the right-down side of the yield CCA plot. This indicated that all of them successfully adapted to low or moderate levels of rain during pre- and post-flowering periods and high-moderate temperature and radiation during post-flowering period as main climate factors driving their performance (Figure 4). Similarly, they also adapted to low levels of organic matter and nutrients and slightly basic pH as the main soil factors determining their yield. Interestingly, the most important factors conditioning yield were also crucial determinants of biomass. This was expected as yield and biomass showed a high and significant correlation (*R* = 0.64, *P* < 0.001). Thus, best performing accessions successfully adapted to high or moderately high temperature and radiation during the post-flowering period and low nutrient content in the soil as crucial factors. Nevertheless, in this case, some of the best performing landraces showed also longer projections over the rain preflowering vector (Figure 4).

Plant height and heading date showed a different trend in the CCA study. Yellow dots represent the tallest and earliest accessions in each panel, which were selected based on their position in the HA-GGE biplot (tallest accessions being those at the right hand side of the height polygon and the earliest, those at the left hand side of the heading polygon, respectively). Briefly, the tallest accessions were between 125 and 150 cm of height, and the earliest accessions headed between 125 to 140 days. As stated above, height and earliness were correlated with a higher oat performance. Figure 4 shows that the tallest landraces projected either near the origin or along the phosphorous and radiation preflowering vectors, whereas the earliest flowering accessions showed high positive projections onto the rain preflowering vector and onto the organic matter, potassium, and nitrogen vectors. Interestingly the landraces with lowest grain abortion grouped very tightly along the rain preflowering vector, which showed a high collinearity with the cation exchange capacity vector.

### Effect of Flowering Time on Climate and Edaphic Factors Determining Yield

As stated above, yield was highly and significantly but negatively correlated with heading date. This was expected as several previous works reported that early flowering might allow cereal plants escaping terminal drought, characteristic of Mediterranean area, favoring production (Shavrukov et al., 2017). As shown above, environmental variables were the main determinants of the variation observed for heading date, and so the effect of these variables on heading date might mask further effect of climatic factors on yield performance. To remove the effect of flowering time from the effect of the environmental variables determining yield, we performed partitioning variation analysis for accessions grouped according to their heading date. The difference in heading date between accessions belonging to the same heading date group did not exceed 10 days considering all replications and environments. Interestingly, for the medium and late flowering groups, the environmental variables explained a proportion of the observed yield variation similar to that explained previously with the complete models. However, the proportion of variation explained by the climate and soil variables was different for the early flowering group (Table 3).

**TABLE 3:** Variance partitioning based on redundancy analysis of yield grouping the different accessions according to the flowering time.

		Partitioning
Early flowering	Climate + interaction	20.43%**
	Soil + interaction	19.09%**
	All	34.26%**
	Climate	15.17% ns
	Soil	13.86% ns
	Interaction	5.26%
Medium flowering	Climate + interaction	42.84%***
	Soil + interaction	19.61%*
	All	52.95%***
	Climate	33.34%*
	Soil	10.11% ns
	Interaction	9.50%
Late flowering	Climate + interaction	44.52%***
	Soil + interaction	22.38%**
	All	53.54%**
	Climate	30.66%*
	Soil	9.2% ns
	Interaction	13.86%

*, **, *** indicate the significance of factors highlighted by the model at P<0.05, 0.1, and 0.001, respectively; ns, non significant differences.

Thus, the proportion of variation explained by climate factors was reduced by 20% in the early flowering group, whereas the proportion of variation explained by edaphic factors remained similar for all groups. Although the overall proportion of variation explained in the early flowering group was reduced, the number of significant variables explaining this variation was the highest (Table 4), suggesting similar importance of most climate variables in the performance of early flowering oats. Although soil characteristic explained a similar fraction of the variation in the three different flowering groups, phosphorous content and organic matter were more determinant for yield in the early flowering group, whereas potassium, organic matter, and cation exchange capacity were more important for the medium and late flowering groups. In particular, the importance of soil cation exchange capacity increased with heading date. Indeed, this variable was not significant for the early flowering group, but was significant in the partial model for chemical characteristics for the medium-flowering group and in all models for the late flowering group (Table 4).

**TABLE 4:** Redundancy analysis of yield grouping the different accessions according to the flowering time.

	Climate					Soil		
		All	Pre	Post		All	Nutrient	Chem
Early flowering	Rain Pre	*	*		N			
	Rad pre		**		P	**	**	
	Aver Tem Pre	*	*		K			
	Rain Post	*		*	OM			**
	Rad post	**		*	C Ex			
	Aver Tem Post			*	pH			
	Model	*P* < 0.001	*P* = 0.006	*P* < 0,001	Model	*P* = 0.018	*P* = 0.017	*P* = 0.009
Medium flowering	Rain Pre		**		N			
	Rad pre		**		P			
	Aver Tem Pre	*	*		K	*	*	
	Rain Post	*		**	OM			
	Rad post			**	C Ex			*
	Aver Tem Post	**			pH			
	Model	*P* < 0.001	*P* < 0.001	*P* < 0.001	Model	*P* = 0.011	*P* = 0.016	*P* = 0.032
Late flowering	Rain Pre		**		N			
	Rad pre	**	**		P			
	Aver Tem Pre				K		**	
	Rain Post				OM	*		*
	Rad post			**	C Ex	*		*
	Aver Tem Post	**		**	pH			
	Model	*P* < 0.001	*P* = 0.003	*P* < 0.001	Model	*P* = 0.003	*P* = 0.004	*P* = 0.007

*, **, indicate the significant factors highlighted by the model at P < 0.05, 0.1, respectively. The significance of the model is also indicated. Ns, non-significant differences.

## Discussion

During last decades, “elite” cultivars have replaced local varieties of many crops reducing their genetic diversity (revised by Newton et al., 2010). These modern cultivars are developed for high yield (Lasa et al., 2001; Gepts and Papa, 2002). However, most oat cultivars assessed in this work showed lower yield and biomass when compared with several landraces. One explanation might be that although modern cultivars are seldom targeted for a specific location, landraces were developed during long-term traditional cultivation at the same location and exposed to both human selection and ecogeographic pressure. Hence they are adapted to fit that environment (Chalak et al., 2015). Thus, under mild environmental cultivation conditions or high input systems, modern cultivars may offer very high yields. However, in low input systems and harsher environments, i.e., marginal soils, drought, or cold prone areas, landraces may outyield cultivars, as we observed.

This is the case of the oat crop in Mediterranean area. Although oat originated from the Fertile Crescent, it developed as a primary crop in the northern regions and consequently is better adapted to cool-temperate environments than to the warmer and drier Mediterranean climate (Thomas, 1995). Despite this, during the last 20 years oat has been increasingly cultivated in the Mediterranean area for its good adaptation to a wide range of soils, in particular in poor soils, because under a low input system oat may perform better than wheat or barley. However, the low attention paid by breeders and policy makers in this area resulted in a relatively low yearly genetic gain in yield in comparison to wheat or barley for which significant breeding investments have been made (Prats et al., 2014). Most oat cultivars used in the Mediterranean area are spring cultivars that are bred in northern countries and used in the Mediterranean rim in autumn sowings (Sánchez-Martín et al., 2017; Rispail et al., 2018). Interestingly, our data highlighted four cultivars that performed relatively well under Mediterranean conditions such as, cv. Cassandra, Alcudia, Rapidena, and Precoce Maroc. Not surprisingly, most of them were among the low number of oat cultivars developed in Mediterranean countries. Thus, Rapidena and Precoce Maroc were developed in Spain and Morocco, respectively. As their names suggest, these cultivars are characterized by early heading date, which could facilitate their escape from the terminal drought characteristic of Mediterranean environments, improving their adaptation (Shavrukov et al., 2017). Alcudia is a cultivar breed in France. It is highly resistant to crown rust (Sánchez-Martín et al., 2012), which might have contributed to the high yield observed. Cassandra is a red oat (subsp. byzantine) developed in Greece. Red oats are considered more adapted to Mediterranean area as they entered cultivation from the Western part of the Mediterranean (Loskutov, 2008). This might explain the slight but significant higher performance of red oats for all agronomic traits assessed in the present work. Interestingly, all cultivars that showed good field performance showed early heading date, whereas it was not always true for the landraces, suggesting that landraces possess further local adaptation capabilities beyond drought escape through early flowering.

Redundancy and canonical correspondence analysis together with variance partitioning allowed determining the relative contribution of climate and edaphic factors to the agronomic traits assessed (Legendre, 2008). These canonical ordination methods offer the possibility to incorporate different environmental variables in the analysis as constrains for the ordination (Legendre, 2008). In particular, variation partitioning is used when two or more complementary sets of hypotheses, in this case the effect of climate and soil, can be invoked to explain the variation of an ecological or agronomic variable such as, in our case, yield or biomass. The total variation was partitioned in different fractions among the sets of environmental variables, and these fractions were tested for significance to fully support the conclusions. As far as we know, there are no other works combining climate and soil variables to partition their effects on main agronomic traits. We observed that most agronomic traits, such as yield and heading date showed significant variation partitioning. The largest fraction was explained by climate variables, whereas the variation explained by soil variables was relatively constant for all agronomic traits. It is known that climate factors largely affect agronomic traits, with precipitation being one of the most important factors determining the final performance, albeit the combination of all-weather elements occurring simultaneously can have additive effects (i.e., Vining, 1990). Climate variables greatly determined the distribution of the environments, as observed in Figure 2. The variation explained by climate and soil factors jointly (named “interaction” in Table 1) represented a relatively large fraction in the case of yield. Surprisingly, soil variables alone, without the interaction of climate factors, were not significant for any of the agronomic traits assessed. This could be due to a lower effect of soil as compared with climate. Alternatively, it may reflect the good adaptation of oat to a wide range of soils (Stevens et al., 2004). Nevertheless, the adjusted multivariate redundancy statistic Ra2 (Miller and Farr, 1971; Legendre, 2008), which estimates the contributions of the climate and edaphic variables to the explanation of the agronomic response, was 0.40 in average, indicating that a large fraction of the observed variation was not explained by the considered climate or soil factors. In addition to these abiotic factors, biotic factors, such as crown rust or powdery mildew infection, largely constrain oat yield and are responsible for yield instability (Sánchez-Martín et al., 2011, 2012). In addition to plant pathogens, other biotic, i.e., beneficial interactions with microorganisms, may also affect oat performance. This highlights the complexity of foreseen agronomic traits in changing environments and the need to include different variables that account as much as possible for the variation observed. As expected, grain abortion was the only agronomic trait exclusively affected by climate variables, in particular, high rain preflowering, high temperature, and low rain post-flowering. The high levels of rain pre-flowering would induce a high number of flowers, whereas high temperatures and water deficit during post-flowering might promote the failure in the filling of the grains (Sánchez-Martín et al., 2017).

Graphical representation of climatic and edaphic variables (in relation to the different agronomic traits) allowed the intuitive determination of collinear traits. Some correlations among variables might be expected, for instance the positive collinearity between cation exchange capacity, nutrient, and organic matter content and their negative correlation with phosphorous content and pH. Cation exchange capacity influences soil structure stability, nutrient availability, soil pH, and the soil’s reaction to fertilizers and other ameliorants (Hazelton and Murphy, 2007). Thus, it influences the soil’s ability to hold onto essential nutrients and provides a buffer against soil acidification. In particular, organic matter has a very high cation exchange capacity, so the correlation observed in Figure 4 is not surprising. Interestingly, although temperature post-flowering was highly correlated with radiation, it was not so during the preflowering period. This might be due to the fact that variation of the atmospheric constituents affecting the relationship between radiation and temperature, i.e., cloudiness, relative humidity, rain, and concentration of atmospheric particles, is smaller during the post-flowering periods in the Mediterranean springs characterized by clear skies than during the preflowering periods characterized by winter-covered skies (Atwater and Ball, 1981; Bristow and Campbell, 1984). Surprisingly, several accessions showed differential adaptation to rain levels during pre- or post-flowering period. This differential adaptation has also been reported in sorghum between others, where QTLs for water deficit tolerance during preflowering periods differed from those identified for coping with post-flowering drought (Tuinstra et al., 1996, 1997). Obviously, the different developmental periods involve different metabolic/physiological processes leading to growth and the differential response might indicate local adaptation to particular microclimates and an evolutionary advantage.

Data showed that landraces showing the best performance, in terms of yield and biomass, in the tested environments presented local adaptation to low or moderate levels of rain pre- and post-flowering, high temperature and radiation post-flowering, and poor nutrient content, which are the main characteristic of most oat cultivation fields in the Mediterranean area (Prats et al., 2014). However, although biomass was highly correlated with yield, landraces with the highest biomass showed adaptation to relatively higher levels of rain preflowering than those showing the highest yield. No lodging was observed in the field experiments. Although lodging is an important issue for oat cultivation in northern areas, it is not a usual limitation of the oat crop in Mediterranean environments. This may be at least in part because oat is not cultivated in the most fertile soils (Prats et al., 2014). Since heading date may largely influence yield in areas such as Mediterranean, (with gradual water depletion as plant mature) while being highly influenced by environmental variables, we grouped the accessions according to their flowering time to infer further the effect of environment on yield (removing the effect of climate on heading date). Interestingly, we observed that soil variables contributed similarly to the variation observed in the different groups. However, climate had a lower contribution to the final observed yield in the early flowering oats than in the mid or late flowering groups. This supports the hypothesis that early flowering accessions are less exposed to extreme environmental events, and in this case terminal drought and/or heat (Shavrukov et al., 2017) and hence, their yield were less influenced by variations in climatic variables. On the other hand, although the contribution of cationic exchange capacity on yield was significant in the general and partial models for the late flowering group, it was only significant for the partial model for the mid flowering group and was not significant in the early flowering group. This suggests that contribution of cation exchange capacity to final plant performance is proportional to heading date. Cation exchange capacity is directly correlated with clay content (Parfitt et al., 1995), and hence with water availability (Farrar and Coleman, 1967), and as stated above, they largely determine nutrient availability. Our data would support these claims since higher cation exchange capacity largely influences the performance of the latest flowering oats growing in the Mediterranean conditions, probably due to the capacity of these soils to retain higher water and nutrient levels.

Plant height of the best performing landraces was highly influenced by the level of phosphorous. Phosphorus is an essential element determining plant growth and productivity (Malhotra et al., 2018). As it plays an important role in cell division and cell enlargement (Assuero et al., 2004), its influence on plant height is not strange. Indeed, in maize, phosphorous has been directly correlated with plant height, in particular under low nitrogen levels (Khan et al., 2014) and several phosphorous-inducible QTLs for plant height were found, which in addition were sensitive to climatic conditions (Cai et al., 2012). Accessions with lower grain abortion grouped around the vector defining rain pre-flowering and cation exchange capacity. These data suggest that high level of rain during pre-flowering period (increasing water reservoir) together with soils characterized by high cation exchange capacity (increasing water availability longer) could reduce grain abortion, and that these accessions were particularly adapted to take advantage of these conditions.

Here, we show for the first time the adaptation strategy of a wide range of oat accessions, including cultivars and landraces to Mediterranean environments and decipher the main climate and edaphic factors driving oat performance under Mediterranean conditions. Taking this into account, plant breeding programs might be able to develop varieties adapted to the specific environments (local adaptation) in which they will be grown, and/or farmers could select those varieties better adapted to their particular growing conditions. This is particularly important where the environment itself cannot be improved by agricultural practices, for instance in low input systems such as the oat cultivation in the Mediterranean rim. Another strategy might be the development of new varieties with wider adaptive plasticity or low sensitivity to the different climatic and/or soil factors (Jinks and Pooni, 1984) by selecting the accessions with good performance clustering near the vector origins. This might be especially relevant for plants that have to adapt to changing scenarios such as in the current context of climate change. Local adaptation to particular environmental conditions or adaptive plasticity may be complementary rather than exclusive. In the current climatic change scenario in which episodes of drought and temporal flooding may alternate in Mediterranean environments, breeding for a wider adaptive plasticity may be more effective in terms of time and cost, although it should be combined with local adaptation in the harshest environments to allow a better plant performance.

## Data Availability Statement

The raw data supporting the conclusions of this article will be made available by the authors, without undue reservation.

## Author Contributions

FC made most of the experimental work and data analysis. GM-B performed most of the experimental work. LMG-S contributed in part of the experimental field. FF made part of the data analysis. NR and EP steered the research, designed experiments, and contributed to the interpretation of results and writing of the manuscript. All authors contributed to critical reading and writing.

## Conflict of Interest

The authors declare that the research was conducted in the absence of any commercial or financial relationships that could be construed as a potential conflict of interest.

## Publisher’s Note

All claims expressed in this article are solely those of the authors and do not necessarily represent those of their affiliated organizations, or those of the publisher, the editors and the reviewers. Any product that may be evaluated in this article, or claim that may be made by its manufacturer, is not guaranteed or endorsed by the publisher.

## Footnotes

1
https://datosclima.es/Aemet2013/LocalizacionEstaciones.php

2
https://www.juntadeandalucia.es

## References

[B1] Assuero S. G. Mollier A. Pellerin S. (2004). The decrease in growth of phosphorus-deficient maize leaves is related to a lower cell production. *Plant Cell Environ.* 27 887–895. 10.1111/j.1365-3040.2004.01194.x

[B2] Atwater M. A. Ball J. T. (1981). Effects of clouds on insolation models. *Solar Energy* 27 37–44. 10.1016/0038-092X(81)90018-9

[B3] Bell G. Lechowicz M. J. Waterway M. J. (2000). Environmental heterogeneity and species diversity of forest sedges. *J. Ecol.* 88 67–87. 10.1046/j.1365-2745.2000.00427.x

[B4] Borcard D. Gillet F. Legendre P. (2017). *Numerical ecology with R.* New York, NY: Springer International Publishing, 10.1007/978-3-319-71404-2

[B5] Box E. O. (1996). Plant functional types and climate at the global scale. *J. Vegetat. Sci.* 7 309–320. 10.2307/3236274

[B6] Bristow K. L. Campbell G. S. (1984). On the relationship between incoming solar-radiation and daily maximum and minimum temperature. *Agricult. For. Meteorol.* 31 159–166. 10.1016/0168-1923(84)90017-0

[B7] Burgueño J. Crossa J. Vargas M. (2003). *SAS programs for graphing GE and GGE biplots.* Mexico: CIMMYT.

[B8] Cai H. G. Chu Q. Gu R. L. Yuan L. X. Liu J. C. Zhang X. Z. (2012). Identification of QTLs for plant height, ear height and grain yield in maize (Zea mays L.) in response to nitrogen and phosphorus supply. *Plant Breed.* 131 502–510. 10.1111/j.1439-0523.2012.01963.x

[B9] Canales F. J. Montilla-Bascón G. Bekele W. A. Howarth C. Langdon T. Rispail N. (2021). Population genomics of mediterranean oat (*A. sativa*) reveals high genetic diversity and three loci for heading date. *Theoret. Appl. Genet.* 134 2063–2077. 10.1007/s00122-021-03805-2 33770189 PMC8263550

[B10] Ceccarelli S. (1994). Specific adaptation and breeding for marginal conditions. *Euphytica* 77 205–219. 10.1007/BF02262633

[B11] Ceccarelli S. (2012). Landraces: importance and use in breeding and environmentally friendly agronomic systems. *Trends Plant Sci.* 2012:0103. 10.1079/9781845938512.0103

[B12] Chalak L. M. R. Rizk W. Hmedeh H. Kabalan R. Breidy J. Hamadeh B. (2015). Performance of 50 Lebanese barley landraces (*Hordeum vulgare* L. subsp. *vulgare*) in two locations under rainfed conditions. *Ann. Agricult. Sci.* 60 325–334. 10.1016/j.aoas.2015.11.005

[B13] Dixon P. (2003). VEGAN, a package of R functions for community ecology. *J. Vegetat. Sci.* 14 927–930. 10.1111/j.1654-1103.2003.tb02228.x

[B14] Dwivedi S. L. Ceccarelli S. Blair M. W. Upadhyaya H. D. Are A. K. Ortiz R. (2016). Lancrace germplasm for improving yield and abiotic stress adaptation. *Trends Plant Sci.* 21 31–42. 10.1016/j.tplants.2015.10.012 26559599

[B15] Ehlers W. (1989). Transpiration efficiency of oat. *Agronomy J.* 81 810–817. 10.2134/agronj1989.00021962008100050023x

[B16] FAO (2017). *Food and agriculture data.* Rome: FAO. Available online at: http://faostat.fao.org (accessed February 17, 2018).

[B17] Farrar D. M. Coleman J. D. (1967). The correlation of surface area with other properties of nineteen British clay soils. *Eur. J. Soil Sci.* 18 118–124. 10.1111/j.1365-2389.1967.tb01493.x

[B18] Fernandez-Aparicio M. Flores F. Rubiales D. (2012). Escape and true resistance to crenate broomrape (*Orobanche crenata* Forsk.) in grass pea (*Lathyrus sativus* L.) germplasm. *Field Crops Res.* 125 92–97. 10.1016/j.fcr.2011.09.003

[B19] Flores F. Hybl M. Knudsen J. C. Marget P. Muel F. Nadal S. (2013). Adaptation of spring faba bean types across European climates. *Field Crops Res.* 145 1–9. 10.1016/j.fcr.2013.01.022

[B20] Flores F. Nadal S. Solis I. Winkler J. Sass O. Stoddard F. L. (2012). Faba bean adaptation to autumn sowing under European climates. *Agronomy Sustain. Dev.* 32 727–734. 10.1007/s13593-012-0082-0

[B21] Frison E. A. Cherfas J. Hodgkin T. (2011). Agricultural biodiversity is essential for a sustainable improvement in food and nutrition security. *Sustainability* 3 238–253. 10.3390/su3010238

[B22] Gauch H. G. Piepho H. P. Annicchiarico P. (2008). Statistical analysis of yield trials by AMMI and GGE: Further considerations. *Crop Sci.* 48 866–889. 10.2135/cropsci2007.09.0513 34798789

[B23] Gepts P. Papa R. (2002). *Evolution during domestication. Encyclopedia of life sciences.* London: Nature Publishing Group, 1–7. 10.1038/npg.els.0003071

[B24] Gilissen L. J. W. J. van der Meer I. M. Smulders M. J. M. (2016). Why oats are safe and healthy for celiac disease patients. *Medical Sci.* 4:21. 10.3390/medsci4040021 29083384 PMC5635790

[B25] Hammer Ø Harper D. A. T. Ryan P. D. (2001). PAST: Paleontological statistics software package for education and data analysis. *Palaeontol. Electron.* 4:9.

[B26] Hazelton P. Murphy B. (2007). *Interpreting soil test results: what to do all the numbers mean?.* Collingwood, VIC: CSIRO Publishing, 10.1071/9781486303977

[B27] Henderson C. R. (1975). Best linear unbiased estimation and prediction under a selection model. *Biometrics* 31 423–447. 10.2307/2529430 1174616

[B28] Jinks J. L. Pooni H. S. (1984). “The genetic basis of environmental sensitivity,” in *Proceedings of the Second International Conference on Quantitative Genetics*, eds Weir B. S. E. Goodman E. J. Namkoong M. M. Sunderland G. (Sunderland, MA: Sinauer Associates Inc), 505–522.

[B29] Khan A. Munsif F. Akhtar K. Afridi M. Z. Zahoor, Ahmad Z. (2014). Response of fodder maize to various levels of nitrogen and phosphorus. *Am. J. Plant Sci.* 5:515246. 10.4236/AJPS.2014.515246

[B30] Kneupffer H. Terentyeva I. Hammer K. Kovaleva O. Sato K. (2003). “Ecogeographical diversity – a Vavilovian approach,” in *Diversity in Barley (Hordeum vulgare). Developments in Plant Genetics and Breeding*, eds von Bothmer R. V. H. Kneupffer T. H. (Amsterdam: Elsevier), 53–76. 10.1016/S0168-7972(03)80006-3

[B31] Lasa J. M. Igartua E. Ciudad F. J. Codesal P. Garcia E. V. Gracia M. P. (2001). Morphological and agronomical diversity patterns in the Spanish barley core collection. *Hereditas* 135 217–225. 10.1111/j.1601-5223.2001.00217.x 12152338

[B32] Lasky J. R. Upadhyaya H. D. Ramu P. Deshpande S. Hash C. T. Bonnette J. (2015). Genotype-environment associations in sorghum landraces predict adaptive traits. *Sci. Adv.* 1:e1400218. 10.1126/sciadv.1400218 26601206 PMC4646766

[B33] Leamy L. J. Lee C. R. Song Q. J. Mujacic I. Luo Y. Chen C. Y. (2016). Environmental versus geographical effects on genomic variation in wild soybean (*Glycine soja*) across its native range in northeast Asia. *Ecol. Evolut.* 6 6332–6344. 10.1002/ece3.2351 27648247 PMC5016653

[B34] Legendre P. (2008). Studying beta diversity: ecological variation partitioning by multiple regression and canonical analysis. *J. Plant Ecol.* 1 3–8. 10.1093/jpe/rtm001

[B35] Li J. S. van Bueren E. T. L. Jiggins J. Leeuwis C. (2012). Farmers’ adoption of maize (*Zea mays* L.) hybrids and the persistence of landraces in Southwest China: implications for policy and breeding. *Genet. Resour. Crop Evolut.* 59 1147–1160. 10.1007/s10722-011-9750-1

[B36] Londono D. M. Smulders M. J. M. Visser R. G. F. Gilissen L. Hamer R. J. (2015). Effect of kilning and milling on the dough-making properties of oat flour. *LWT Food Sci. Technol.* 63 960–965. 10.1016/j.lwt.2015.04.033

[B37] Loskutov I. G. (2008). On evolutionary pathways of *Avena* species. *Genet. Resour. Crop Evolut.* 55 211–220. 10.1007/s10722-007-9229-2

[B38] Macel M. Lawson C. S. Mortimer S. R. Smilauerova M. Bischoff A. Cremieux L. (2007). Climate vs. soil factors in local adaptation of two common plant species. *Ecology* 88 424–433. 10.1890/0012-9658(2007)88[424:cvsfil]2.0.co;2 17479760

[B39] Malhotra H. Vandana, Sharma S. Pandey R. (2018). Phosphorus nutrition: plant growth in response to deficiency and excess. *Plant Nutrients Abiotic Stress Tolerance* 2018 171–190. 10.1007/978-981-10-9044-8_7

[B40] Martinez-Villaluenga C. Peñas E. (2017). Health benefits of oat: current evidence and molecular mechanisms. *Curr. Opin. Food Sci.* 14 26–31. 10.1016/j.cofs.2017.01.004

[B41] Miller J. K. Farr S. D. (1971). Bimultivariate redundancy - comprehensive measure of interbattery relationship. *Multivar. Behav. Res.* 6 313–324. 10.1207/s15327906mbr0603_4

[B42] Montilla-Bascón G. Sanchez-Martin J. Rispail N. Rubiales D. Mur L. Langdon T. (2013). Genetic diversity and population structure among oat cultivars and landraces. *Plant Mol. Biol. Rep*. 31 1305–1314.

[B43] Newton A. C. Akar T. Baresel J. P. Bebeli P. J. Bettencourt E. Bladenopoulos K. V. (2010). Cereal landraces for sustainable agriculture. A review. *Agronomy Sustain. Dev.* 30 237–269. 10.1007/978-94-007-0394-0_10

[B44] Oksanen J. Blanchet F. G. Friendly M. Kindt R. Legendre P. McGlinn D. (2020). *vegan: Community Ecology Package. R package version 2.5-7.* Vienna: R Core Team.

[B45] Parfitt R. L. Giltrap D. J. Whitton J. S. (1995). Contribution of organic matter and clay minerals to the cation exchange capacity of soils. *Commun. Soil Sci. Plant Anal.* 26 1343–1355. 10.1080/00103629509369376

[B46] Parsad R. Crossa J. Gupta V. K. Gupta R. K. Ladha J. K. Raman A. (2009). “Statistical tools for farmers’ participatory trials for conservation agriculture,” in *Integrated Crop and Resource Management in the Rice–Wheat System of South Asia*, eds Ladha J. K. Singh Y. Erenstein O. Hardy B. (Los Banõs: International Rice Research Institute), 279–296.

[B47] Prats E. Sánchez-Martín J. Montilla-Bascón G. Rubiales D. Rispail N. (2014). *Overview and perspectives of the oat crop in spain.* Philadelphia, PA: Oat newsletter.

[B48] R Development Core Team (2008). *R: A language and environment for statistical computing.* Vienna: R Foundation for Statistical Computing.

[B49] Rispail N. Montilla-Bascon G. Sanchez-Martin J. Flores F. Howarth C. Langdon T. (2018). Multi-environmental trials reveal genetic plasticity of oat agronomic traits associated with climate variable changes. *Front. Plant Sci.* 9:01358. 10.3389/fpls.2018.01358 30283476 PMC6156136

[B50] Rubiales D. Avila C. M. Sillero J. C. Hybl M. Narits L. Sass O. (2012). Identification and multi-environment validation of resistance to *Ascochyta fabae* in faba bean (*Vicia faba*). *Field Crops Res.* 126 165–170. 10.1016/j.fcr.2011.10.012

[B51] Rubiales D. Flores F. Emeran A. A. Kharrat M. Amri M. Rojas-Molina M. M. (2014). Identification and multi-environment validation of resistance against broomrapes (*Orobanche crenata* and *Orobanche foetida*) in faba bean (*Vicia faba*). *Field Crops Res.* 166 58–65. 10.1016/j.fcr.2014.06.010

[B52] Sánchez-Martín J. Mur L. A. J. Rubiales D. Prats E. (2012). Targeting sources of drought tolerance within an *Avena* spp. collection through multivariate approaches. *Planta* 236 1529–1545. 10.1007/s00425-012-1709-8 22824964

[B53] Sánchez-Martín J. Rispail N. Flores F. Emeran A. A. Sillero J. C. Rubiales D. (2017). Higher rust resistance and similar yield of oat landraces versus cultivars under high temperature and drought. *Agronomy Sustain. Dev.* 37 407–405. 10.1007/s13593-016-0407-5

[B54] Sánchez-Martín J. Rubiales D. Prats E. (2011). Resistance to powdery mildew (*Blumeria graminis* f.sp ***avenae***) in oat seedlings and adult plants. *Plant Pathol.* 60 846–856. 10.1111/j.1365-3059.2011.02453.x

[B55] Sánchez-Martín J. Rubiales D. Flores F. Emeran A. A. Shtaya M. J. Y. Sillero J. C. (2014). Adaptation of oat (*Avena sativa*) cultivars to autumn sowings in Mediterranean environments. *Field Crops Res.* 156 111–122. 10.1016/j.fcr.2013.10.018

[B56] Santamaria L. Figuerola J. Pilon J. J. Mjelde M. Green A. J. De Boer T. (2003). Plant performance across latitude: The role of plasticity and local adaptation in an aquatic plant. *Ecology* 84 2454–2461. 10.1890/02-0431

[B57] Shavrukov Y. Kurishbayev A. Jatayev S. Shvidchenko V. Zotova L. Koekemoer F. (2017). Early flowering as a drought escape mechanism in plants: how can it aid wheat production? *Front. Plant Sci.* 8:01950. 10.3389/fpls.2017.01950 29204147 PMC5698779

[B58] Stevens E. J. Armstrong K. W. Bezar H. J. Griffin W. B. Hampton J. G. (2004). “Fodder oats: an overview,” in *Fodder oats: A world overview*, eds Suttie J. M. Reynolds S. G. (Rome: FAO), 1–9.

[B59] Thomas H. (1995). “Oats,” in *Evolution of crop plants*, eds Smartt J. Simmonds N. (London: Longman), 133–137.

[B60] Tuinstra M. R. Grote E. M. Goldsbrough P. B. Ejeta G. (1996). Identification of quantitative trait loci associated with pre-flowering drought tolerance in sorghum. *Crop Sci.* 36 1337–1344. 10.2135/cropsci1996.0011183X003600050043x 34798789

[B61] Tuinstra M. R. Grote E. M. Goldsbrough P. B. Ejeta G. (1997). Genetic analysis of post-flowering drought tolerance and components of grain development in *Sorghum bicolor* (L.) Moench. *Mol. Breed.* 3 439–448. 10.1023/A:1009673126345

[B62] van der Putten W. H. de Ruiter P. C. Bezemer T. M. Harvey J. A. Wassen M. Wolters V. (2004). Trophic interactions in a changing world. *Basic Appl. Ecol.* 5 487–494. 10.1016/j.baae.2004.09.003

[B63] Villegas-Fernandez A. M. Sillero J. C. Emeran A. A. Winkler J. Raffiot B. Tay J. (2009). Identification and multi-environment validation of resistance to Botrytis fabae in Vicia faba. *Field Crops Res.* 114 84–90. 10.1016/j.fcr.2009.07.005

[B64] Vining K. C. (1990). Effects of weather on agricultural crops and livestock: an overview. *Int. J. Environ. Stud.* 36 27–39. 10.1080/00207239008710581

[B65] Wardle D. A. (2002). *Communities and ecosystems: linking the aboveground and belowground components.* Princeton, NJ: Princeton University Press, 10.1515/9781400847297

[B66] Williams G. C. (1966). *Adaptation and natural selection.* Princeton, NJ: Princeton University Press, 10.1093/oso/9780198829539.003.0007

[B67] Yahiaoui S. Cuesta-Marcos A. Gracia M. P. Medina B. Lasa J. M. Casas A. M. (2014). Spanish barley landraces outperform modern cultivars at low-productivity sites. *Plant Breed.* 133 218–226. 10.1111/pbr.12148

[B68] Yan W. K. Holland J. B. (2010). A heritability-adjusted GGE biplot for test environment evaluation. *Euphytica* 171 355–369. 10.1007/s10681-009-0030-5

[B69] Yang R. C. Crossa J. Cornelius P. L. Burgueño J. (2009). Biplot analysis of genotype × environment interaction: proceed with caution. *Crop Sci.* 49 1564–1576. 10.2135/cropsci2008.11.0665 34798789

[B70] Zar J. H. (1984). *Biostatistical analysis.* Englewood Cliffs: Prentice-Hall, Inc.

