# Supplementary material for: Deciphering Main Climate and Edaphic Components Driving Oat Adaptation to Mediterranean Environments
Source: Front Plant Sci. 2021 Nov 26;12:780562. doi: 10.3389/fpls.2021.780562 (PMC8662754; doi:10.3389/fpls.2021.780562)
Supplement: Supplementary file 1 [file Data_Sheet_1.pdf]

Frontiers in Plant Science

Supplemental Material

Deciphering main climate and edaphic components driving oat adaptation to Mediterranean environments

Francisco J Canales[1], Gracia Montilla-Bascón[1], Luis M. Gallego-Sánchez[1], Fernando Flores[2], Nicolas Rispail[1], Elena Prats[1]

[1]*CSIC, Institute of Sustainable Agriculture, Córdoba, Spain.*

[2]*E.T.S.I. El Carmen, Univ. Huelva, 21007 Huelva, Spain*.

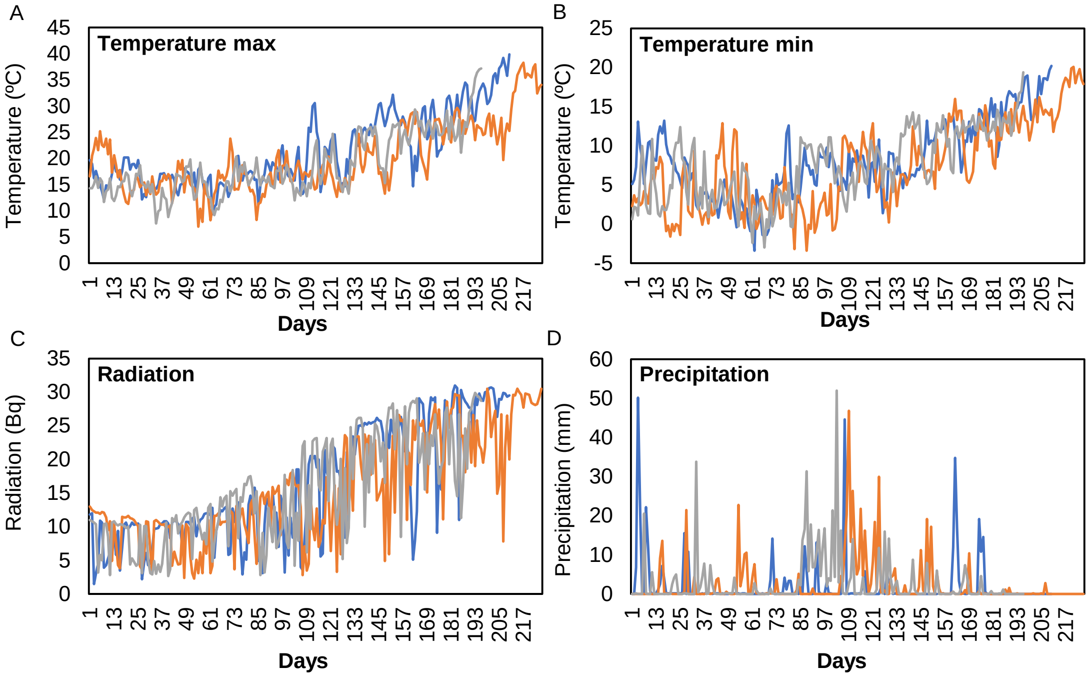

Supplemental Figure 1. Climate variables including (A) maximum and (B) minimum temperatures, (C) radiation and (D) precipitation during the field experiments at different environments: Cordoba 2016/2017 (blue line), Cordoba 2017-2018 (orange line) and Santaella 2017/2018 (gray line).

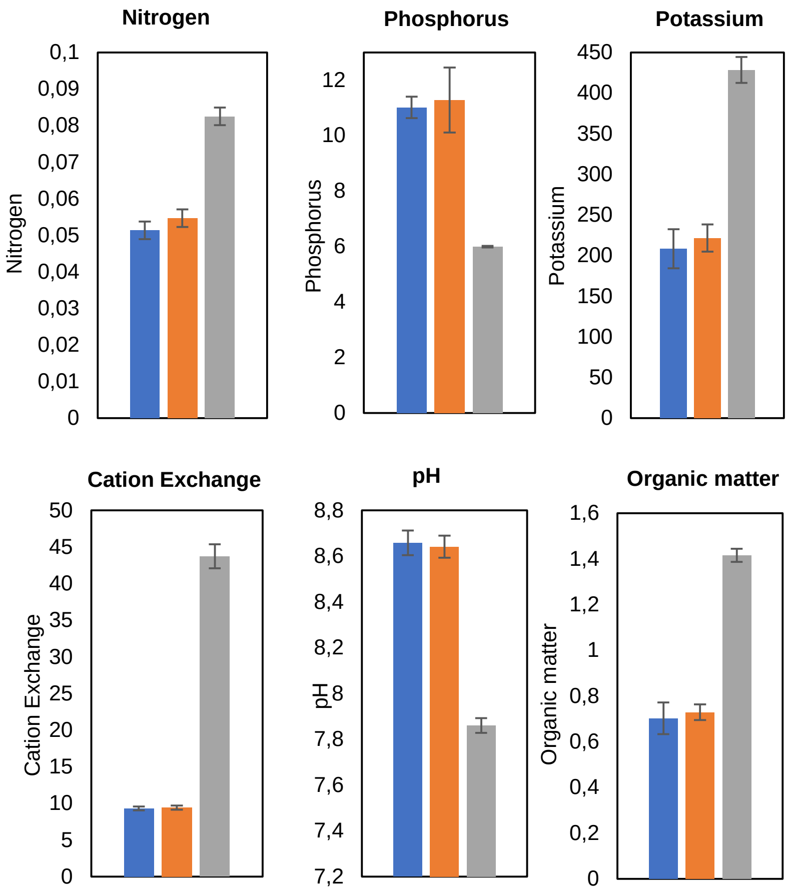

Supplemental Figure 2. Soil variables including nitrogen, phosphorous, potassium, cation exchange, pH and organic matter recorded during the field experiments in the different environments: CO17 (blue bars), CO18 (orange bars) and SA18 (gray bars). Data are mean of 3 replicates + standard error**.**

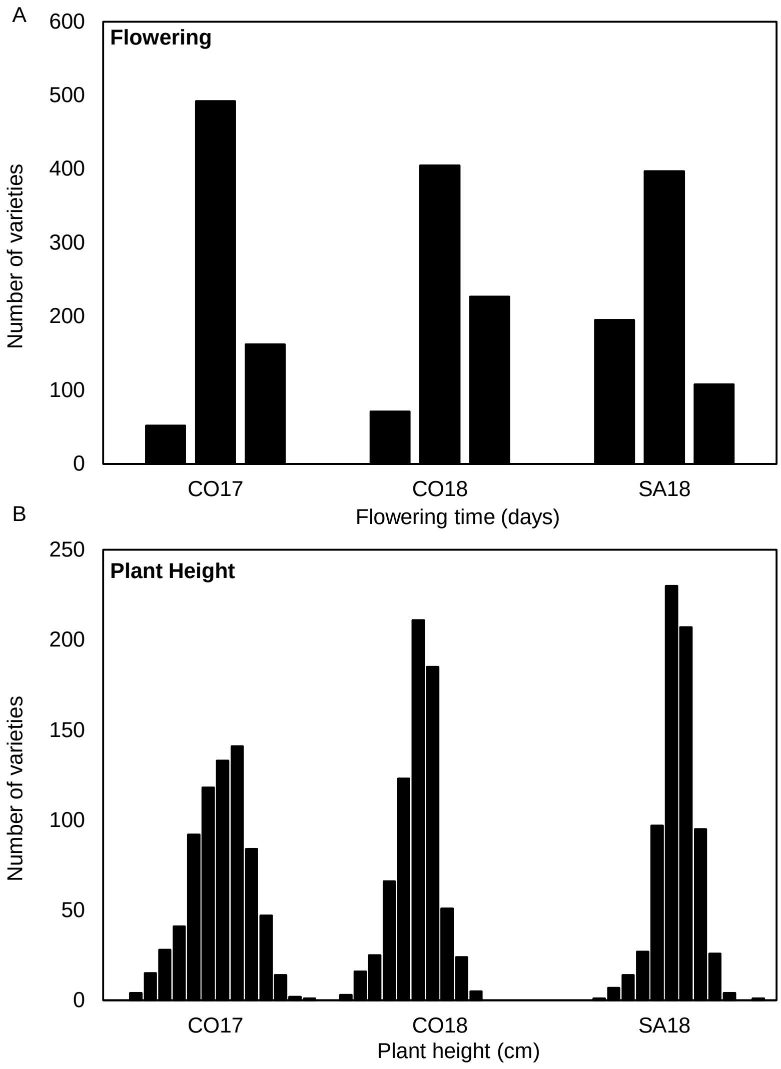

Supplemental Figure 3. Histograms of heading date and plant height the three environments CO17, CO18 and SA18, respectively. Histograms for heading date shows the early, mid and late flowering groups ranging from 125 to 181 days and those of height ranging from 99 to 173 cm.

Supplemental Table 1. Heading date and height of the oat collection used in this study. Data are means of three different environments.

name	Bank code	Species	Subspecies (STRUCTURE clasification)	Heading date (days)	Plant Height (cm)
**AV.IAS001**	BGE008113	Avena sativa	Sativa	170	109
**AV.IAS002**	BGE008115	Avena sativa	Sativa	161	149
**AV.IAS003**	BGE008116	Avena sativa	Sativa	168	138
**AV.IAS004**	BGE008117	Avena sativa	Sativa	145	135
**AV.IAS005**	BGE008118	Avena sativa	Sativa	146	139
**AV.IAS006**	BGE008119	Avena sativa	Sativa	144	115
**AV.IAS007**	BGE008121	Avena sativa	Sativa	145	148
**AV.IAS008**	BGE008122	Avena sativa	Sativa	169	133
**AV.IAS009**	BGE008123	Avena sativa	Sativa	144	116
**AV.IAS010**	BGE008124	Avena sativa	Sativa	151	138
**AV.IAS011**	BGE008140	Avena sativa	Byzantina	148	136
**AV.IAS012**	BGE008174	Avena sativa	Sativa	168	112
**AV.IAS013**	BGE008175	Avena sativa	Sativa	150	144
**AV.IAS014**	BGE008176	Avena sativa	Sativa	162	96
**AV.IAS015**	BGE008177	Avena sativa	Sativa	144	135
**AV.IAS016**	BGE008178	Avena sativa	Sativa	146	165
**AV.IAS017**	BGE008179	Avena sativa	Sativa	160	146
**AV.IAS018**	BGE008180	Avena sativa	Sativa	166	132
**AV.IAS019**	BGE008189	Avena sativa	Sativa	145	125
**AV.IAS020**	BGE008200	Avena sativa	Sativa	158	147
**AV.IAS021**	BGE008201	Avena sativa	Sativa	147	103
**AV.IAS022**	BGE008202	Avena sativa	Sativa	177	132
**AV.IAS023**	BGE008279	Avena sativa	Sativa	145	157
**AV.IAS024**	BGE008382	Avena sativa	Byzantina	148	112
**AV.IAS025**	BGE008397	Avena sativa	Byzantina	141	132
**AV.IAS026**	BGE008398	Avena sativa	Byzantina	149	109
**AV.IAS027**	BGE008399	Avena sativa	Sativa	165	106
**AV.IAS028**	BGE008400	Avena sativa	Byzantina	148	123
**AV.IAS029**	BGE008401	Avena sativa	Sativa	141	129
**AV.IAS030**	BGE008402	Avena sativa	Sativa	150	127
**AV.IAS031**	BGE008403	Avena sativa	Sativa	153	116
**AV.IAS032**	BGE008404	Avena sativa	Sativa	142	127
**AV.IAS033**	BGE008405	Avena sativa	Sativa	145	124
**AV.IAS034**	BGE008406	Avena sativa	Sativa	144	119
**AV.IAS035**	BGE008407	Avena sativa	Sativa	161	134
**AV.IAS036**	BGE008408	Avena sativa	Sativa	172	123
**AV.IAS037**	BGE008409	Avena sativa	Sativa	156	123
**AV.IAS038**	BGE008477	Avena sativa	Byzantina	144	142
**AV.IAS039**	BGE008478	Avena sativa	Sativa	157	127
**AV.IAS040**	BGE008479	Avena sativa	Sativa	173	115
**AV.IAS041**	BGE008480	Avena sativa	Sativa	145	142
**AV.IAS042**	BGE008481	Avena sativa	Sativa	170	124
**AV.IAS043**	BGE008679	Avena sativa	Sativa	171	117
**AV.IAS044**	BGE009393	Avena sativa	Sativa	150	135
**AV.IAS045**	BGE009394	Avena sativa	Byzantina	149	130

name	Bank code	Species	Subspecies (STRUCTURE clasification)	Heading date (days)	Plant Height (cm)
**AV.IAS046**	BGE009395	Avena sativa	Sativa	144	134
**AV.IAS047**	BGE009631	Avena sativa	Sativa	162	117
**AV.IAS048**	BGE009632	Avena sativa	Sativa	173	111
**AV.IAS049**	BGE009633	Avena sativa	Sativa	158	143
**AV.IAS050**	BGE009634	Avena sativa		148	146
**AV.IAS051**	BGE009642	Avena sativa	Sativa	167	127
**AV.IAS052**	BGE009643	Avena sativa	Sativa	160	136
**AV.IAS053**	BGE009644	Avena sativa	Sativa	162	111
**AV.IAS054**	BGE009645	Avena sativa	Sativa	162	112
**AV.IAS055**	BGE009646	Avena sativa	Sativa	144	123
**AV.IAS056**	BGE009647	Avena sativa	Sativa	144	131
**AV.IAS057**	BGE009648	Avena sativa	Sativa	142	141
**AV.IAS058**	BGE009649	Avena sativa	Sativa	147	150
**AV.IAS059**	BGE009668	Avena sativa	Sativa	143	133
**AV.IAS060**	BGE009711	Avena sativa	Sativa	172	104
**AV.IAS061**	BGE009721	Avena sativa	Sativa	176	146
**AV.IAS062**	BGE009722	Avena sativa	Sativa	169	122
**AV.IAS063**	BGE009723	Avena sativa	Sativa	171	125
**AV.IAS064**	BGE009746	Avena sativa	Sativa	163	108
**AV.IAS065**	BGE009754	Avena sativa	Byzantina	150	137
**AV.IAS066**	BGE009748	Avena sativa	Sativa	174	131
**AV.IAS067**	BGE009772	Avena sativa	Byzantina	150	137
**AV.IAS068**	BGE010416	Avena sativa	Byzantina	150	136
**AV.IAS069**	BGE010418	Avena sativa	Sativa	162	111
**AV.IAS070**	BGE010419	Avena sativa	Sativa	170	144
**AV.IAS071**	BGE010420	Avena sativa	Sativa	173	103
**AV.IAS072**	BGE010421	Avena sativa	Byzantina	150	117
**AV.IAS073**	BGE010423	Avena sativa	Sativa	158	142
**AV.IAS074**	BGE010424	Avena sativa	Sativa	146	138
**AV.IAS075**	BGE010425	Avena sativa	Sativa	173	131
**AV.IAS076**	BGE010426	Avena sativa	Byzantina	145	94
**AV.IAS077**	BGE010427	Avena sativa	Sativa	161	129
**AV.IAS078**	BGE010428	Avena sativa	Sativa	158	152
**AV.IAS079**	BGE010429	Avena sativa	Byzantina	147	162
**AV.IAS080**	BGE010430	Avena sativa	Byzantina	147	139
**AV.IAS081**	BGE010431	Avena sativa	Sativa	166	116
**AV.IAS082**	BGE010432	Avena sativa	Sativa	168	118
**AV.IAS083**	BGE010433	Avena sativa	Sativa	171	106
**AV.IAS084**	BGE010434	Avena sativa	Sativa	144	116
**AV.IAS085**	BGE010498	Avena sativa	Sativa	158	145
**AV.IAS086**	BGE010499	Avena sativa	Sativa	144	126
**AV.IAS087**	BGE010500	Avena sativa	Sativa	155	127
**AV.IAS088**	BGE010501	Avena sativa	Sativa	147	150
**AV.IAS089**	BGE010502	Avena sativa	Sativa	147	157
**AV.IAS090**	BGE010503	Avena sativa	Sativa	141	122
**AV.IAS091**	BGE010504	Avena sativa	Sativa	143	115
**AV.IAS092**	BGE010505	Avena sativa	Sativa	160	139
**AV.IAS093**	BGE015353	Avena sativa	Sativa	142	123

name	Bank code	Species	Subspecies (STRUCTURE clasification)	Heading date (days)	Plant Height (cm)
**AV.IAS094**	BGE015354	Avena sativa	Sativa	143	110
**AV.IAS095**	BGE015355	Avena sativa	Byzantina	152	136
**AV.IAS096**	BGE015356	Avena sativa	Byzantina	152	135
**AV.IAS097**	BGE015357	Avena sativa		143	121
**AV.IAS098**	BGE015358	Avena sativa	Sativa	158	118
**AV.IAS099**	BGE015359	Avena sativa	Byzantina	145	137
**AV.IAS100**	BGE015360	Avena sativa	Byzantina	147	173
**AV.IAS101**	BGE018448	Avena sativa	Sativa	177	138
**AV.IAS102**	BGE018452	Avena sativa	Sativa	159	120
**AV.IAS103**	BGE018453	Avena sativa	Sativa	145	136
**AV.IAS104**	BGE018454	Avena sativa	Sativa	173	118
**AV.IAS105**	BGE018455	Avena sativa	Sativa	160	131
**AV.IAS106**	BGE018482	Avena sativa	Sativa	174	141
**AV.IAS107**	BGE018483	Avena sativa	Sativa	171	123
**AV.IAS108**	BGE018484	Avena sativa	Sativa	170	126
**AV.IAS109**	BGE018485	Avena sativa	Byzantina	147	143
**AV.IAS110**	BGE018486	Avena sativa	Byzantina	150	121
**AV.IAS111**	BGE018487	Avena sativa	Sativa	158	139
**AV.IAS112**	BGE018488	Avena sativa	Sativa	145	118
**AV.IAS113**	BGE018489	Avena sativa	Sativa	164	126
**AV.IAS114**	BGE018490	Avena sativa	Sativa	158	131
**AV.IAS115**	BGE018491	Avena sativa	Sativa	169	121
**AV.IAS116**	BGE018492	Avena sativa	Sativa	156	146
**AV.IAS117**	BGE018495	Avena sativa	Sativa	156	131
**AV.IAS118**	BGE018498	Avena sativa	Sativa	158	119
**AV.IAS119**	BGE018499	Avena sativa	Sativa	151	132
**AV.IAS120**	BGE018501	Avena sativa	Sativa	146	146
**AV.IAS121**	BGE018567	Avena sativa	Sativa	146	135
**AV.IAS122**	BGE018568	Avena sativa	Sativa	144	128
**AV.IAS123**	BGE018569	Avena sativa	Sativa	143	129
**AV.IAS124**	BGE018570	Avena sativa	Sativa	141	122
**AV.IAS125**	BGE018576	Avena sativa	Byzantina	153	125
**AV.IAS126**	BGE018577	Avena sativa	Byzantina	153	138
**AV.IAS127**	BGE018579	Avena sativa	Byzantina	157	131
**AV.IAS128**	BGE018580	Avena sativa	Byzantina	151	144
**AV.IAS129**	BGE018581	Avena sativa	Byzantina	151	138
**AV.IAS130**	BGE018582	Avena sativa	Byzantina	150	133
**AV.IAS131**	BGE018583	Avena sativa	Byzantina	150	137
**AV.IAS132**	BGE018584	Avena sativa	Byzantina	149	100
**AV.IAS133**	BGE018585	Avena sativa	Admixture	161	139
**AV.IAS134**	BGE018588	Avena sativa	Byzantina	147	158
**AV.IAS135**	BGE018591	Avena sativa	Byzantina	151	133
**AV.IAS136**	BGE018593	Avena sativa	Sativa	157	137
**AV.IAS137**	BGE020322	Avena sativa	Sativa	157	130
**AV.IAS138**	BGE026963	Avena sativa	Sativa	147	110
**AV.IAS139**	BGE026964	Avena sativa	Sativa	147	147
**AV.IAS140**	BGE026965	Avena sativa	Sativa	160	141
**AV.IAS141**	BGE030934	Avena sativa	Sativa	162	117

name	Bank code	Species	Subspecies (STRUCTURE clasification)	Heading date (days)	Plant Height (cm)
**AV.IAS142**	BGE005494	Avena sativa	Byzantina	145	138
**AV.IAS143**	BGE005495	Avena sativa	Sativa	181	137
**AV.IAS144**	BGE008130	Avena sativa	Sativa	162	114
**AV.IAS145**	BGE008131	Avena sativa	Sativa	160	134
**AV.IAS146**	BGE008142	Avena sativa	Sativa	148	144
**AV.IAS147**	BGE008281	Avena sativa	Sativa	167	111
**AV.IAS148**	BGE008410	Avena sativa	Sativa	155	139
**AV.IAS149**	BGE008625	Avena sativa	Sativa	150	137
**AV.IAS150**	BGE008626	Avena sativa	Sativa	148	125
**AV.IAS151**	BGE008627	Avena sativa	Sativa	166	121
**AV.IAS152**	BGE008628	Avena sativa	Sativa	174	117
**AV.IAS153**	BGE008629	Avena sativa	Sativa	165	119
**AV.IAS154**	BGE008630	Avena sativa	Sativa	162	111
**AV.IAS155**	BGE008631	Avena sativa	Sativa	148	140
**AV.IAS156**	BGE008632	Avena sativa	Sativa	175	148
**AV.IAS157**	BGE008633	Avena sativa	Sativa	173	130
**AV.IAS158**	BGE008634	Avena sativa	Sativa	165	126
**AV.IAS159**	BGE008635	Avena sativa	Sativa	167	124
**AV.IAS160**	BGE008636	Avena sativa	Sativa	163	119
**AV.IAS161**	BGE008637	Avena sativa	Sativa	176	141
**AV.IAS162**	BGE008638	Avena sativa	Sativa	169	116
**AV.IAS163**	BGE008639	Avena sativa	Sativa	172	111
**AV.IAS164**	BGE008640	Avena sativa	Sativa	164	111
**AV.IAS165**	BGE008641	Avena sativa	Sativa	168	136
**AV.IAS166**	BGE008642	Avena sativa	Sativa	169	117
**AV.IAS167**	BGE008643	Avena sativa	Sativa	167	137
**AV.IAS168**	BGE008644	Avena sativa	Sativa	143	123
**AV.IAS169**	BGE008645	Avena sativa	Sativa	163	141
**AV.IAS170**	BGE008646	Avena sativa	Sativa	144	119
**AV.IAS171**	BGE008647	Avena sativa	Byzantina	154	128
**AV.IAS172**	BGE008648	Avena sativa	Byzantina	151	138
**AV.IAS173**	BGE008649	Avena sativa	Byzantina	150	122
**AV.IAS174**	BGE008650	Avena sativa	Sativa	147	129
**AV.IAS175**	BGE008651	Avena sativa	Byzantina	147	136
**AV.IAS176**	BGE008652	Avena sativa		151	128
**AV.IAS177**	BGE008653	Avena sativa	Sativa	169	133
**AV.IAS178**	BGE008654	Avena sativa	Sativa	143	124
**AV.IAS179**	BGE008655	Avena sativa	Sativa	142	126
**AV.IAS180**	BGE008656	Avena sativa	Sativa	168	126
**AV.IAS181**	BGE008657	Avena sativa	Sativa	146	159
**AV.IAS182**	BGE008658	Avena sativa	Sativa	163	109
**AV.IAS183**	BGE008659	Avena sativa	Sativa	167	121
**AV.IAS184**	BGE008660	Avena sativa	Sativa	164	109
**AV.IAS185**	BGE009448	Avena sativa	Sativa	166	117
**AV.IAS186**	BGE009449	Avena sativa	Sativa	166	128
**AV.IAS187**	BGE009450	Avena sativa	Sativa	170	133
**AV.IAS188**	BGE009451	Avena sativa	Sativa	166	113
**AV.IAS189**	BGE009452	Avena sativa	Sativa	169	131

name	Bank code	Species	Subspecies (STRUCTURE clasification)	Heading date (days)	Plant Height (cm)
**AV.IAS190**	BGE009453	Avena sativa	Sativa	169	132
**AV.IAS191**	BGE009454	Avena sativa	Sativa	170	124
**AV.IAS192**	BGE009455	Avena sativa	Sativa	166	126
**AV.IAS193**	BGE009456	Avena sativa	Sativa	173	117
**AV.IAS194**	BGE009457	Avena sativa	Sativa	170	124
**AV.IAS195**	BGE009672	Avena sativa	Sativa	175	145
**AV.IAS196**	BGE009675	Avena sativa	Sativa	166	128
**AV.IAS197**	BGE009684	Avena sativa	Sativa	166	113
**AV.IAS198**	BGE009736	Avena sativa	Byzantina	139	124
**AV.IAS199**	BGE009749	Avena sativa	Byzantina	150	121
**AV.IAS200**	BGE009750	Avena sativa	Byzantina	143	118
**AV.IAS201**	BGE009753	Avena sativa	Byzantina	147	136
**AV.IAS202**	BGE009757	Avena sativa	Sativa	159	143
**AV.IAS203**	BGE009758	Avena sativa	Byzantina	146	142
**AV.IAS204**	BGE009759	Avena sativa	Byzantina	147	153
**AV.IAS205**	BGE009760	Avena sativa	Byzantina	146	123
**AV.IAS206**	BGE009767	Avena sativa	Byzantina	142	128
**AV.IAS207**	BGE009773	Avena sativa	Byzantina	139	125
**AV.IAS208**	BGE009774	Avena sativa	Sativa	163	151
**AV.IAS209**	BGE015361	Avena sativa	Byzantina	147	140
**AV.IAS210**	BGE015362	Avena sativa	Sativa	145	132
**AV.IAS211**	BGE015363	Avena sativa	Sativa	153	125
**AV.IAS212**	BGE018424	Avena sativa	Sativa	164	139
**AV.IAS213**	BGE018425	Avena sativa	Sativa	167	133
**AV.IAS214**	BGE018427	Avena sativa	Sativa	167	160
**AV.IAS215**	BGE018428	Avena sativa	Sativa	167	113
**AV.IAS216**	BGE018442	Avena sativa	Sativa	170	120
**AV.IAS217**	BGE018443	Avena sativa	Sativa	173	125
**AV.IAS218**	BGE018444	Avena sativa	Sativa	164	107
**AV.IAS219**	BGE018462	Avena sativa	Sativa	177	133
**AV.IAS220**	BGE018463	Avena sativa	Sativa	176	159
**AV.IAS221**	BGE018464	Avena sativa	Sativa	173	116
**AV.IAS222**	BGE018465	Avena sativa	Sativa	178	136
**AV.IAS223**	BGE018466	Avena sativa		166	134
**AV.IAS224**	BGE018467	Avena sativa	Sativa	155	152
**AV.IAS225**	BGE018468	Avena sativa	Sativa	169	131
**AV.IAS226**	BGE018469	Avena sativa	Sativa	170	118
**AV.IAS227**	BGE018470	Avena sativa	Sativa	174	104
**AV.IAS228**	BGE018471	Avena sativa	Sativa	172	118
**AV.IAS229**	BGE018472	Avena sativa	Sativa	174	121
**AV.IAS230**	BGE018473	Avena sativa	Sativa	175	137
**AV.IAS231**	BGE018474	Avena sativa	Sativa	178	142
**AV.IAS232**	BGE018475	Avena sativa		154	143
**AV.IAS233**	BGE018572	Avena sativa	Admixture	141	126
**AV.IAS234**	BGE018573	Avena sativa	Sativa	168	117
**AV.IAS235**	BGE018574	Avena sativa	Sativa	160	150
**AV.IAS236**	BGE018575	Avena sativa	Byzantina	152	136
**AV.IAS237**	BGE018589	Avena sativa	Sativa	149	124

name	Bank code	Species	Subspecies (STRUCTURE clasification)	Heading date (days)	Plant Height (cm)
**AV.IAS238**	BGE018590	Avena sativa	Byzantina	151	124
**AV.IAS239**	BGE019839	Avena sativa	Sativa	162	100
**AV.IAS240**	BGE019840	Avena sativa	Sativa	159	146
**AV.IAS241**	BGE020323	Avena sativa	Sativa	171	142
**AV.IAS242**	BGE023978	Avena sativa	Byzantina	153	115
**AV.IAS243**	BGE026966	Avena sativa	Sativa	173	116
**AV.IAS244**	BGE026968	Avena sativa	Byzantina	147	139
**AV.IAS245**	BGE042532	Avena sativa	Sativa	154	140
**AV.IAS246**	BGE042533	Avena sativa	Byzantina	147	147
**AV.IAS247**	Clav 1822	Avena sativa	Sativa	158	133
**AV.IAS248**	PI 253347	Avena sativa	Byzantina	140	101
**AV.IAS249**	PI 287274	Avena sativa	Byzantina	163	124
**AV.IAS250**	PI 287278	Avena sativa	Byzantina	162	88
**AV.IAS251**	PI 287279	Avena sativa	Byzantina	165	122
**AV.IAS252**	PI 287280	Avena sativa	Byzantina	164	135
**AV.IAS253**	PI 287306	Avena sativa	Byzantina	151	132
**AV.IAS254**	PI 287338	Avena sativa	Sativa	166	109
**AV.IAS255**	PI 287353	Avena sativa	Sativa	162	100
**AV.IAS256**	PI 287354	Avena sativa	Sativa	162	78
**AV.IAS257**	PI 287441	Avena sativa	Sativa	161	122
**AV.IAS258**	PI 287443	Avena sativa		163	115
**AV.IAS259**	Clav 1832	Avena sativa	Byzantina	161	144
**AV.IAS260**	Clav 2033	Avena sativa	Byzantina	151	137
**AV.IAS261**	PI 10269	Avena sativa	Sativa	149	101
**AV.IAS262**	PI 46565	Avena sativa	Byzantina	152	146
**AV.IAS263**	PI 48089	Avena sativa	Sativa	156	146
**AV.IAS264**	PI 57043	Avena sativa		148	122
**AV.IAS265**	Clav 2332	Avena sativa	Byzantina	149	128
**AV.IAS266**	Clav 2333	Avena sativa	Byzantina	148	159
**AV.IAS267**	Clav 9082	Avena sativa	Sativa	160	166
**AV.IAS268**	Clav 9102	Avena sativa	Byzantina	153	118
**AV.IAS269**	Clav 9103	Avena sativa	Byzantina	152	148
**AV.IAS270**	PI 158205	Avena sativa	Byzantina	143	116
**AV.IAS271**	PI 258557	Avena sativa	Byzantina	150	143
**AV.IAS272**	PI 258558	Avena sativa	Byzantina	147	141
**AV.IAS273**	PI 258559	Avena sativa	Byzantina	150	129
**AV.IAS274**	PI 258582	Avena sativa	Byzantina	151	145
**AV.IAS275**	PI 258583	Avena sativa	Byzantina	150	129
**AV.IAS276**	PI 263412	Avena sativa	Byzantina	151	128
**AV.IAS277**	PI 264211	Avena sativa	Byzantina	152	130
**AV.IAS278**	PI 264212	Avena sativa	Byzantina	151	134
**AV.IAS279**	PI 573570	Avena sativa	Byzantina	151	132
**AV.IAS280**	PI 158206	Avena sativa	Byzantina	152	127
**AV.IAS281**	PI 158212	Avena sativa	Byzantina	137	127
**AV.IAS282**	PI 258547	Avena sativa	Byzantina	149	123
**AV.IAS283**	PI 258549	Avena sativa	Byzantina	148	151
**AV.IAS284**	PI 374390	Avena sativa	Sativa	171	123
**AV.IAS285**	PI 374394	Avena sativa	Sativa	159	127

name	Bank code	Species	Subspecies (STRUCTURE clasification)	Heading date (days)	Plant Height (cm)
**AV.IAS286**	PI 374396	Avena sativa	Sativa	159	131
**AV.IAS287**	PI 374397	Avena sativa	Sativa	164	113
**AV.IAS288**	PI 374400	Avena sativa	Sativa	171	125
**AV.IAS289**	PI 374406	Avena sativa	Sativa	173	149
**AV.IAS290**	PI 374407	Avena sativa	Sativa	159	129
**AV.IAS291**	PI 378198	Avena sativa	Sativa	168	122
**AV.IAS292**	PI 378199	Avena sativa	Sativa	163	131
**AV.IAS293**	PI 378200	Avena sativa	Sativa	168	120
**AV.IAS294**	PI 378201	Avena sativa	Sativa	168	136
**AV.IAS295**	PI 378207	Avena sativa	Sativa	161	138
**AV.IAS296**	PI 378208	Avena sativa	Sativa	161	125
**AV.IAS297**	PI 378209	Avena sativa	Sativa	160	139
**AV.IAS298**	PI 378250	Avena sativa	Sativa	160	102
**AV.IAS299**	Clav 7251	Avena sativa	Sativa	158	128
**AV.IAS300**	PI 158231	Avena sativa	Sativa	161	138
**AV.IAS301**	PI 158240	Avena sativa	Sativa	175	146
**AV.IAS302**	PI 184019	Avena sativa	Sativa	159	143
**AV.IAS303**	PI 251575	Avena sativa	Sativa	169	118
**AV.IAS304**	PI 251576	Avena sativa	Sativa	164	120
**AV.IAS305**	PI 251577	Avena sativa	Sativa	156	136
**AV.IAS306**	PI 251578	Avena sativa	Sativa	171	109
**AV.IAS307**	PI 251579	Avena sativa	Sativa	169	131
**AV.IAS308**	PI 251581	Avena sativa	Sativa	168	135
**AV.IAS309**	PI 251582	Avena sativa	Sativa	170	128
**AV.IAS310**	PI 253296	Avena sativa	Sativa	165	110
**AV.IAS311**	PI 253297	Avena sativa	Sativa	173	128
**AV.IAS312**	PI 264857	Avena sativa	Sativa	170	114
**AV.IAS313**	PI 266274	Avena sativa	Sativa	165	125
**AV.IAS314**	PI 344818	Avena sativa	Sativa	158	143
**AV.IAS315**	PI 344819	Avena sativa	Sativa	162	122
**AV.IAS316**	PI 344820	Avena sativa	Sativa	155	145
**AV.IAS317**	PI 344821	Avena sativa	Sativa	156	138
**AV.IAS318**	PI 344822	Avena sativa	Sativa	159	141
**AV.IAS319**	PI 344823	Avena sativa	Sativa	170	117
**AV.IAS320**	PI 344824	Avena sativa	Sativa	169	127
**AV.IAS321**	PI 344825	Avena sativa	Sativa	171	114
**AV.IAS322**	PI 344826	Avena sativa	Sativa	159	143
**AV.IAS323**	PI 344832	Avena sativa	Sativa	170	122
**AV.IAS324**	PI 349854	Avena sativa	Sativa	159	142
**AV.IAS325**	PI 374408	Avena sativa	Sativa	172	136
**AV.IAS326**	PI 158238	Avena sativa	Sativa	166	117
**AV.IAS327**	PI 184000	Avena sativa	Sativa	167	147
**AV.IAS328**	PI 184013	Avena sativa	Sativa	164	115
**AV.IAS329**	PI 184021	Avena sativa	Sativa	158	141
**AV.IAS330**	PI 184022	Avena sativa	Sativa	161	136
**AV.IAS331**	PI 184023	Avena sativa	Sativa	154	132
**AV.IAS332**	PI 184024	Avena sativa	Sativa	169	127
**AV.IAS333**	PI 184025	Avena sativa	Sativa	168	128

name	Bank code	Species	Subspecies (STRUCTURE clasification)	Heading date (days)	Plant Height (cm)
**AV.IAS334**	PI 222502	Avena sativa	Sativa	175	119
**AV.IAS335**	PI 222503	Avena sativa	Sativa	168	131
**AV.IAS336**	PI 222504	Avena sativa	Sativa	172	118
**AV.IAS337**	PI 222505	Avena sativa	Sativa	159	122
**AV.IAS338**	PI 259865	Avena sativa	Sativa	155	122
**AV.IAS339**	PI 259866	Avena sativa	Sativa	155	131
**AV.IAS340**	PI 259867	Avena sativa	Sativa	171	131
**AV.IAS341**	PI 259868	Avena sativa	Sativa	172	134
**AV.IAS342**	PI 259869	Avena sativa	Sativa	175	134
**AV.IAS343**	PI 259870	Avena sativa	Sativa	164	134
**AV.IAS344**	PI 259871	Avena sativa	Sativa	171	129
**AV.IAS345**	PI 259872	Avena sativa	Sativa	164	133
**AV.IAS346**	PI 259873	Avena sativa	Sativa	170	112
**AV.IAS347**	PI 259874	Avena sativa	Admixture	150	141
**AV.IAS348**	PI 264858	Avena sativa	Sativa	155	127
**AV.IAS349**	PI 264859	Avena sativa	Sativa	161	136
**AV.IAS350**	PI 264860	Avena sativa	Sativa	162	112
**AV.IAS351**	PI 264861	Avena sativa	Sativa	165	135
**AV.IAS352**	PI 269185	Avena sativa	Sativa	177	130
**AV.IAS353**	PI 269189	Avena sativa	Sativa	169	138
**AV.IAS354**	PI 287396	Avena sativa	Sativa	176	130
**AV.IAS355**	PI 287397	Avena sativa	Sativa	171	149
**AV.IAS356**	PI 287398	Avena sativa	Sativa	176	141
**AV.IAS357**	PI 287399	Avena sativa	Sativa	163	122
**AV.IAS358**	PI 189756	Avena sativa	Sativa	149	117
**AV.IAS359**	PI 365614	Avena sativa	Sativa	153	125
**AV.IAS360**	PI 365615	Avena sativa	Sativa	150	137
**AV.IAS361**	PI 365616	Avena sativa	Sativa	148	136
**AV.IAS362**	PI 365617	Avena sativa	Sativa	154	138
**AV.IAS363**	PI 365619	Avena sativa	Sativa	148	146
**AV.IAS364**	PI 365620	Avena sativa	Sativa	160	138
**AV.IAS365**	PI 365621	Avena sativa	Sativa	150	126
**AV.IAS366**	PI 365622	Avena sativa	Sativa	152	164
**AV.IAS367**	PI 249721	Avena sativa	Byzantina	161	139
**AV.IAS368**	PI 249932	Avena sativa	Byzantina	145	141
**AV.IAS369**	PI 249933	Avena sativa	Byzantina	145	122
**AV.IAS370**	Clav 357	Avena sativa	Sativa	159	143
**AV.IAS371**	Clav 9100	Avena sativa	Byzantina	156	137
**AV.IAS372**	PI 219760	Avena sativa	Byzantina	163	148
**AV.IAS373**	PI 219761	Avena sativa	Byzantina	145	129
**AV.IAS374**	PI 219762	Avena sativa	Byzantina	163	122
**AV.IAS375**	PI 219763	Avena sativa	Byzantina	164	108
**AV.IAS376**	PI 219764	Avena sativa	Byzantina	159	138
**AV.IAS377**	PI 219765	Avena sativa	Byzantina	157	125
**AV.IAS378**	PI 258566	Avena sativa	Byzantina	149	141
**AV.IAS379**	PI 258579	Avena sativa	Byzantina	153	129
**AV.IAS380**	PI 258580	Avena sativa	Byzantina	145	118
**AV.IAS381**	PI 258584	Avena sativa	Byzantina	160	147

name	Bank code	Species	Subspecies (STRUCTURE clasification)	Heading date (days)	Plant Height (cm)
**AV.IAS382**	PI 258585	Avena sativa	Byzantina	149	142
**AV.IAS383**	PI 264427	Avena sativa	Byzantina	144	127
**AV.IAS384**	PI 264428	Avena sativa	Byzantina	144	133
**AV.IAS385**	PI 264429	Avena sativa	Byzantina	162	113
**AV.IAS386**	PI 264838	Avena sativa	Byzantina	149	126
**AV.IAS387**	PI 264839	Avena sativa	Byzantina	157	143
**AV.IAS388**	PI 264840	Avena sativa	Byzantina	159	137
**AV.IAS389**	PI 264841	Avena sativa	Byzantina	160	169
**AV.IAS390**	PI 264842	Avena sativa	Byzantina	159	152
**AV.IAS391**	PI 264843	Avena sativa	Byzantina	157	142
**AV.IAS392**	PI 264844	Avena sativa	Byzantina	153	153
**AV.IAS393**	PI 264845	Avena sativa	Byzantina	151	136
**AV.IAS394**	PI 264846	Avena sativa	Byzantina	157	102
**AV.IAS395**	PI 264847	Avena sativa	Byzantina	156	127
**AV.IAS396**	PI 264848	Avena sativa	Byzantina	151	134
**AV.IAS397**	PI 264849	Avena sativa	Sativa	158	136
**AV.IAS398**	PI 264850	Avena sativa	Sativa	162	91
**AV.IAS399**	PI 264851	Avena sativa	Byzantina	163	129
**AV.IAS400**	PI 264854	Avena sativa	Byzantina	164	114
**AV.IAS401**	PI 264862	Avena sativa	Admixture	145	137
**AV.IAS402**	PI 264863	Avena sativa	Byzantina	155	142
**AV.IAS403**	PI 287264	Avena sativa	Byzantina	147	133
**AV.IAS404**	PI 287265	Avena sativa	Byzantina	157	146
**AV.IAS405**	PI 287266	Avena sativa	Byzantina	151	137
**AV.IAS406**	PI 287267	Avena sativa	Byzantina	140	107
**AV.IAS407**	PI 287269	Avena sativa	Byzantina	145	109
**AV.IAS408**	PI 287270	Avena sativa	Byzantina	162	85
**AV.IAS409**	PI 287271	Avena sativa	Byzantina	163	97
**AV.IAS410**	PI 287272	Avena sativa	Byzantina	157	127
**AV.IAS411**	PI 287273	Avena sativa	Byzantina	165	119
**AV.IAS412**	PI 287275	Avena sativa	Byzantina	167	123
**AV.IAS413**	PI 287276	Avena sativa	Byzantina	163	112
**AV.IAS414**	PI 287277	Avena sativa	Byzantina	162	111
**AV.IAS415**	PI 287281	Avena sativa	Byzantina	163	122
**AV.IAS416**	PI 287282	Avena sativa	Byzantina	158	139
**AV.IAS417**	PI 287283	Avena sativa	Byzantina	161	138
**AV.IAS418**	PI 287284	Avena sativa	Byzantina	146	147
**AV.IAS419**	PI 287285	Avena sativa	Byzantina	146	132
**AV.IAS420**	PI 287286	Avena sativa	Byzantina	160	139
**AV.IAS421**	PI 287287	Avena sativa	Byzantina	158	132
**AV.IAS422**	PI 287288	Avena sativa	Byzantina	151	139
**AV.IAS423**	PI 287289	Avena sativa	Byzantina	157	133
**AV.IAS424**	PI 287290	Avena sativa	Byzantina	159	148
**AV.IAS425**	PI 287292	Avena sativa	Byzantina	158	134
**AV.IAS426**	PI 287300	Avena sativa	Byzantina	158	137
**AV.IAS427**	PI 287301	Avena sativa	Byzantina	158	126
**AV.IAS428**	PI 287302	Avena sativa	Byzantina	156	143
**AV.IAS429**	PI 287303	Avena sativa	Byzantina	156	134

name	Bank code	Species	Subspecies (STRUCTURE clasification)	Heading date (days)	Plant Height (cm)
**AV.IAS430**	PI 287304	Avena sativa	Byzantina	162	101
**AV.IAS431**	PI 287305	Avena sativa	Byzantina	161	120
**AV.IAS432**	PI 287336	Avena sativa	Sativa	148	136
**AV.IAS433**	PI 287337	Avena sativa	Sativa	146	132
**AV.IAS434**	PI 287339	Avena sativa	Sativa	156	127
**AV.IAS435**	PI 287341	Avena sativa	Sativa	160	142
**AV.IAS436**	PI 287351	Avena sativa	Sativa	153	132
**AV.IAS437**	PI 287380	Avena sativa	Sativa	162	114
**AV.IAS438**	PI 287442	Avena sativa	Sativa	164	119
**AV.IAS439**	PI 287461	Avena sativa	Sativa	158	142
**AV.IAS440**	PI 287462	Avena sativa	Sativa	167	121
**AV.IAS441**	PI 338516	Avena sativa	Byzantina	148	156
**AV.IAS442**	PI 338517	Avena sativa	Sativa	155	123
**AV.IAS443**	PI 201989	Avena sativa	Byzantina	153	135
**AV.IAS444**	PI 295953	Avena sativa	Sativa	165	128
**AV.IAS445**	PI 295954	Avena sativa	Sativa	151	142
**AV.IAS446**	PI 295955	Avena sativa	Sativa	161	129
**AV.IAS447**	PI 298126	Avena sativa	Byzantina	140	117
**AV.IAS448**	Clav 7653	Avena sativa	Sativa	156	103
**AV.IAS449**	Clav 8090	Avena sativa	Sativa	162	81
**AV.IAS450**	PI 253501	Avena sativa	Sativa	148	137
**AV.IAS451**	PI 291989	Avena sativa	Byzantina	144	112
**AV.IAS452**	PI 573568	Avena sativa	Byzantina	154	97
**AV.IAS453**	PI 157879	Avena sativa	Sativa	159	151
**AV.IAS454**	PI 61999	Avena sativa	Sativa	170	146
**AV.IAS455**	PI 110254	Avena sativa	Sativa	156	132
**AV.IAS456**	PI 110255	Avena sativa	Byzantina	147	151
**AV.IAS457**	PI 110257	Avena sativa	Sativa	170	122
**AV.IAS458**	PI 110258	Avena sativa	Sativa	156	133
**AV.IAS459**	PI 110259	Avena sativa	Byzantina	147	141
**AV.IAS460**	PI 110260	Avena sativa	Sativa	155	123
**AV.IAS461**	PI 198908	Avena sativa	Sativa	160	134
**AV.IAS462**	PI 564724	Avena sativa	Byzantina	145	127
**AV.IAS463**	PI 564725	Avena sativa	Admixture	137	150
**AV.IAS464**	Clav 2864	Avena sativa	Sativa	160	141
**AV.IAS465**	Clav 2866	Avena sativa	Sativa	149	144
**AV.IAS466**	PI 137598	Avena sativa	Byzantina	148	128
**AV.IAS467**	PI 157878	Avena sativa	Byzantina	150	117
**AV.IAS468**	PI 157880	Avena sativa	Sativa	164	132
**AV.IAS469**	PI 157881	Avena sativa	Admixture	146	134
**AV.IAS470**	PI 157882	Avena sativa	Byzantina	145	103
**AV.IAS471**	PI 157883	Avena sativa	Byzantina	152	137
**AV.IAS472**	PI 221276	Avena sativa		153	129
**AV.IAS473**	PI 221277	Avena sativa	Sativa	160	133
**AV.IAS474**	PI 258548	Avena sativa	Byzantina	150	138
**AV.IAS475**	PI 258569	Avena sativa	Byzantina	144	127
**AV.IAS476**	PI 258570	Avena sativa	Byzantina	152	136
**AV.IAS477**	PI 287293	Avena sativa	Byzantina	154	155

name	Bank code	Species	Subspecies (STRUCTURE clasification)	Heading date (days)	Plant Height (cm)
**AV.IAS478**	PI 287294	Avena sativa	Byzantina	153	143
**AV.IAS479**	PI 287295	Avena sativa	Byzantina	153	135
**AV.IAS480**	PI 287296	Avena sativa	Byzantina	152	147
**AV.IAS481**	PI 287297	Avena sativa	Byzantina	150	126
**AV.IAS482**	PI 287298	Avena sativa	Byzantina	148	134
**AV.IAS483**	PI 287307	Avena sativa	Byzantina	150	134
**AV.IAS484**	PI 264206	Avena sativa	Sativa	153	142
**AV.IAS485**	PI 264207	Avena sativa	Byzantina	128	120
**AV.IAS486**	PI 344827	Avena sativa	Sativa	170	131
**AV.IAS487**	PI 344828	Avena sativa	Sativa	168	145
**AV.IAS488**	PI 344829	Avena sativa	Sativa	176	121
**AV.IAS489**	PI 344830	Avena sativa	Sativa	168	125
**AV.IAS490**	PI 344831	Avena sativa	Sativa	172	121
**AV.IAS491**	PI 344833	Avena sativa	Sativa	155	117
**AV.IAS492**	PI 344834	Avena sativa	Sativa	166	137
**AV.IAS493**	PI 344835	Avena sativa	Sativa	162	96
**AV.IAS494**	PI 344836	Avena sativa	Sativa	164	121
**AV.IAS495**	PI 344849	Avena sativa	Sativa	161	107
**AV.IAS496**	PI 349858	Avena sativa	Sativa	159	143
**AV.IAS497**	PI 349859	Avena sativa	Sativa	162	113
**AV.IAS498**	PI 349878	Avena sativa	Sativa	167	154
**AV.IAS499**	PI 349879	Avena sativa	Sativa	169	128
**AV.IAS500**	PI 362363	Avena sativa	Sativa	168	136
**AV.IAS501**	PI 362364	Avena sativa	Sativa	176	137
**AV.IAS502**	PI 362366	Avena sativa	Sativa	175	140
**AV.IAS503**	PI 362367	Avena sativa	Sativa	166	127
**AV.IAS504**	PI 362373	Avena sativa	Sativa	160	130
**AV.IAS505**	PI 321759	Avena sativa	Sativa	156	136
**AV.IAS506**	PI 321760	Avena sativa	Sativa	160	138
**AV.IAS507**	PI 321761	Avena sativa	Sativa	160	132
**AV.IAS508**	PI 321762	Avena sativa	Sativa	165	114
**AV.IAS509**	PI 321763	Avena sativa	Sativa	153	134
**AV.IAS510**	PI 321764	Avena sativa	Sativa	155	130
**AV.IAS511**	PI 321765	Avena sativa	Sativa	165	123
**AV.IAS512**	PI 321766	Avena sativa	Sativa	162	107
**AV.IAS513**	PI 321767	Avena sativa	Sativa	169	129
**AV.IAS514**	PI 158208	Avena sativa	Admixture	166	122
**AV.IAS515**	PI 258564	Avena sativa	Byzantina	160	137
**AV.IAS516**	PI 258565	Avena sativa	Byzantina	155	124
**AV.IAS517**	PI 266266	Avena sativa	Byzantina	145	128
**AV.IAS518**	PI 411397	Avena sativa	Sativa	152	146
**AV.IAS519**	PI 573569	Avena sativa	Byzantina	143	115
**AV.IAS520**	PI 55521	Avena sativa	Admixture	149	134
**AV.IAS521**	PI 55523	Avena sativa	Byzantina	152	137
**AV.IAS522**	PI 55524	Avena sativa	Byzantina	146	138
**AV.IAS523**	PI 158213	Avena sativa	Byzantina	142	138
**AV.IAS524**	PI 189766	Avena sativa	Byzantina	148	164
**AV.IAS525**	PI 189767	Avena sativa	Byzantina	151	127

name	Bank code	Species	Subspecies (STRUCTURE clasification)	Heading date (days)	Plant Height (cm)
**AV.IAS526**	PI 258560	Avena sativa	Sativa	159	116
**AV.IAS527**	PI 258561	Avena sativa	Byzantina	153	129
**AV.IAS528**	PI 258563	Avena sativa	Byzantina	151	111
**AV.IAS529**	PI 411398	Avena sativa	Byzantina	147	152
**AV.IAS530**	Clav 9023	Avena sativa	Byzantina	131	122
**AV.IAS531**	Clav 9045	Avena sativa	Byzantina	144	126
**AV.IAS532**	PI 158211	Avena sativa	Byzantina	151	126
**AV.IAS533**	PI 258567	Avena sativa	Byzantina	155	143
**AV.IAS534**	PI 258568	Avena sativa	Byzantina	153	152
**AV.IAS535**	PI 258581	Avena sativa	Byzantina	160	123
**AV.IAS536**	PI 266265	Avena sativa	Byzantina	153	131
**AV.IAS537**	PI 293344	Avena sativa	Sativa	142	126
**AV.IAS538**	PI 293345	Avena sativa	Sativa	132	123
**AV.IAS539**	PI 293346	Avena sativa	Byzantina	129	127
**AV.IAS540**	PI 293347	Avena sativa	Byzantina	148	145
**AV.IAS541**	PI 293348	Avena sativa	Sativa	141	128
**AV.IAS542**	PI 573572	Avena sativa	Byzantina	160	124
**AV.IAS543**	Clav 1073	Avena sativa	Sativa	164	141
**AV.IAS544**	Clav 1261	Avena sativa	Sativa	160	123
**AV.IAS545**	Clav 1346	Avena sativa	Sativa	152	140
**AV.IAS546**	Clav 1606	Avena sativa	Sativa	168	122
**AV.IAS547**	Clav 2167	Avena sativa	Sativa	164	119
**AV.IAS548**	Clav 5079	Avena sativa	Sativa	157	102
**AV.IAS549**	Clav 2166	Avena sativa	Sativa	161	125
**AV.IAS550**	Clav 5078	Avena sativa	Sativa	161	143
**AV.IAS551**	Clav 5080	Avena sativa	Sativa	164	112
**AV.IAS552**	Clav 5081	Avena sativa	Sativa	168	121
**AV.IAS553**	PI 235166	Avena sativa	Sativa	176	126
**AV.IAS554**	PI 401783	Avena sativa	Sativa	160	154
**AV.IAS555**	PI 119474	Avena sativa	Sativa	165	111
**AV.IAS556**	PI 119476	Avena sativa	Sativa	154	160
**AV.IAS557**	PI 119477	Avena sativa	Sativa	156	135
**AV.IAS558**	PI 119478	Avena sativa	Sativa	156	150
**AV.IAS559**	PI 168067	Avena sativa	Byzantina	151	132
**AV.IAS560**	PI 168068	Avena sativa	Sativa	164	114
**AV.IAS561**	PI 168069	Avena sativa	Byzantina	149	108
**AV.IAS562**	PI 168070	Avena sativa	Byzantina	153	153
**AV.IAS563**	PI 168071	Avena sativa	Byzantina	153	124
**AV.IAS564**	PI 168072	Avena sativa	Admixture	161	93
**AV.IAS565**	PI 168073	Avena sativa	Byzantina	159	127
**AV.IAS566**	PI 168074	Avena sativa	Byzantina	145	143
**AV.IAS567**	PI 168075	Avena sativa	Byzantina	144	140
**AV.IAS568**	PI 168077	Avena sativa	Byzantina	149	133
**AV.IAS569**	PI 168078	Avena sativa	Byzantina	150	126
**AV.IAS570**	PI 168079	Avena sativa	Byzantina	152	142
**AV.IAS571**	PI 168080	Avena sativa	Byzantina	149	121
**AV.IAS572**	PI 168081	Avena sativa	Sativa	152	125
**AV.IAS573**	PI 168082	Avena sativa	Sativa	159	142

name	Bank code	Species	Subspecies (STRUCTURE clasification)	Heading date (days)	Plant Height (cm)
**AV.IAS574**	PI 168083	Avena sativa	Sativa	155	146
**AV.IAS575**	PI 168088	Avena sativa	Byzantina	162	111
**AV.IAS576**	PI 168089	Avena sativa	Byzantina	162	129
**AV.IAS577**	PI 168090	Avena sativa	Byzantina	159	139
**AV.IAS578**	PI 168091	Avena sativa	Byzantina	163	89
**AV.IAS579**	PI 168092	Avena sativa	Byzantina	159	141
**AV.IAS580**	PI 168096	Avena sativa	Byzantina	163	100
**AV.IAS581**	PI 168097	Avena sativa	Byzantina	160	149
**AV.IAS582**	PI 168098	Avena sativa	Byzantina	157	142
**AV.IAS583**	PI 168099	Avena sativa	Byzantina	163	139
**AV.IAS584**	PI 168101	Avena sativa	Byzantina	161	128
**AV.IAS585**	PI 168102	Avena sativa	Sativa	149	98
**AV.IAS586**	PI 168103	Avena sativa	Byzantina	161	129
**AV.IAS587**	PI 168104	Avena sativa	Byzantina	162	115
**AV.IAS588**	PI 168105	Avena sativa	Byzantina	161	135
**AV.IAS589**	PI 168106	Avena sativa	Byzantina	151	144
**AV.IAS590**	PI 168107	Avena sativa	Sativa	158	112
**AV.IAS591**	PI 168108	Avena sativa	Sativa	156	143
**AV.IAS592**	PI 168109	Avena sativa	Sativa	150	118
**AV.IAS593**	PI 168110	Avena sativa	Sativa	160	153
**AV.IAS594**	PI 168111	Avena sativa	Byzantina	158	123
**AV.IAS595**	PI 168112	Avena sativa	Sativa	172	124
**AV.IAS596**	PI 168113	Avena sativa	Sativa	164	124
**AV.IAS597**	PI 168114	Avena sativa	Sativa	149	138
**AV.IAS598**	PI 168115	Avena sativa	Sativa	158	156
**AV.IAS599**	PI 168116	Avena sativa	Sativa	155	151
**AV.IAS600**	PI 168118	Avena sativa	Byzantina	148	139
**AV.IAS601**	PI 168119	Avena sativa	Sativa	168	119
**AV.IAS602**	PI 168121	Avena sativa	Sativa	161	119
**AV.IAS603**	PI 168122	Avena sativa	Sativa	145	131
**AV.IAS604**	PI 168123	Avena sativa	Sativa	159	137
**AV.IAS605**	PI 168124	Avena sativa	Sativa	159	140
**AV.IAS606**	PI 168126	Avena sativa	Sativa	162	87
**AV.IAS607**	PI 170243	Avena sativa	Byzantina	142	129
**AV.IAS608**	PI 170244	Avena sativa	Byzantina	153	138
**AV.IAS609**	PI 170935	Avena sativa	Sativa	159	134
**AV.IAS610**	PI 170936	Avena sativa	Sativa	151	129
**AV.IAS611**	PI 170937	Avena sativa	Sativa	162	96
**AV.IAS612**	PI 172499	Avena sativa	Byzantina	153	138
**AV.IAS613**	PI 173579	Avena sativa	Sativa	146	148
**AV.IAS614**	PI 173585	Avena sativa	Sativa	152	112
**AV.IAS615**	PI 177766	Avena sativa	Byzantina	149	114
**AV.IAS616**	PI 177767	Avena sativa	Byzantina	150	128
**AV.IAS617**	PI 177768	Avena sativa	Byzantina	152	157
**AV.IAS618**	PI 177770	Avena sativa	Byzantina	160	132
**AV.IAS619**	PI 177771	Avena sativa	Byzantina	159	137
**AV.IAS620**	PI 177772	Avena sativa	Byzantina	161	126
**AV.IAS621**	PI 177773	Avena sativa	Byzantina	166	136

name	Bank code	Species	Subspecies (STRUCTURE clasification)	Heading date (days)	Plant Height (cm)
**AV.IAS622**	PI 177818	Avena sativa	Byzantina	158	129
**AV.IAS623**	PI 177821	Avena sativa	Byzantina	159	135
**AV.IAS624**	PI 177824	Avena sativa	Byzantina	153	136
**AV.IAS625**	PI 177825	Avena sativa	Byzantina	154	136
**AV.IAS626**	PI 177840	Avena sativa	Byzantina	156	133
**AV.IAS627**	PI 177841	Avena sativa	Byzantina	165	141
**AV.IAS628**	PI 177842	Avena sativa	Byzantina	158	143
**AV.IAS629**	PI 177843	Avena sativa	Byzantina	163	109
**AV.IAS630**	PI 177844	Avena sativa	Byzantina	170	141
**AV.IAS631**	PI 177845	Avena sativa	Byzantina	161	152
**AV.IAS632**	PI 177846	Avena sativa	Byzantina	162	113
**AV.IAS633**	PI 177848	Avena sativa	Byzantina	159	154
**AV.IAS634**	PI 177855	Avena sativa	Byzantina	160	124
**AV.IAS635**	PI 177859	Avena sativa	Sativa	153	138
**AV.IAS636**	PI 177860	Avena sativa	Sativa	154	148
**AV.IAS637**	PI 177861	Avena sativa	Sativa	148	117
**AV.IAS638**	PI 177862	Avena sativa	Sativa	152	136
**AV.IAS639**	PI 203450	Avena sativa	Sativa	156	137
**AV.IAS640**	PI 406699	Avena sativa	Byzantina	152	113
**AV.IAS641**	PI 406700	Avena sativa	Sativa	145	132
**AV.IAS642**	PI 577953	Avena sativa	Sativa	150	134
**AV.IAS643**	Clav 9101	Avena sativa	Byzantina	125	146
**AV.IAS644**	PI 158228	Avena sativa	Sativa	160	118
**AV.IAS645**	PI 166969	Avena sativa	Byzantina	150	128
**AV.IAS646**	PI 167227	Avena sativa	Byzantina	149	140
**AV.IAS647**	PI 167280	Avena sativa	Byzantina	150	133
**AV.IAS648**	PI 178474	Avena sativa	Byzantina	158	131
**AV.IAS649**	PI 178479	Avena sativa	Byzantina	151	131
**AV.IAS650**	PI 258575	Avena sativa	Byzantina	150	135
**AV.IAS651**	PI 266972	Avena sativa	Byzantina	160	152
**AV.IAS652**	PI 340989	Avena sativa	Byzantina	156	127
**AV.IAS653**	PI 340990	Avena sativa	Sativa	174	130
**AV.IAS654**	PI 341011	Avena sativa	Sativa	160	139
**AV.IAS655**	PI 341014	Avena sativa	Sativa	153	129
**AV.IAS656**	PI 411400	Avena sativa	Byzantina	157	136
**AV.IAS657**	PI 411409	Avena sativa	Sativa	154	130
**AV.IAS658**	PI 411411	Avena sativa	Sativa	147	140
**AV.IAS659**	PI 411412	Avena sativa	Byzantina	161	133
**AV.IAS660**	PI 411415	Avena sativa	Sativa	157	127
**AV.IAS661**	PI 411417	Avena sativa	Byzantina	159	144
**AV.IAS662**	PI 411421	Avena sativa	Byzantina	158	141
**AV.IAS663**	PI 411426	Avena sativa	Sativa	154	143
**AV.IAS664**	PI 411427	Avena sativa	Sativa	160	134
**AV.IAS665**	Clav 9104	Avena sativa	Byzantina	146	160
**AV.IAS666**	Clav 9106	Avena sativa	Admixture	162	111
**AV.IAS667**	PI 152437	Avena sativa	Byzantina	140	106
**AV.IAS668**	PI 152438	Avena sativa	Admixture	132	144
**AV.IAS669**	PI 258562	Avena sativa	Byzantina	157	132

name	Bank code	Species	Subspecies (STRUCTURE clasification)	Heading date (days)	Plant Height (cm)
AV.IAS670	NA	Avena sativa	Sativa	166	123
AV.IAS671	NA	Avena sativa	Sativa	133	132
AV.IAS672	NA	Avena sativa	Sativa	157	131
AV.IAS673	NA	Avena sativa	Sativa	163	126
AV.IAS674	NA	Avena sativa	Sativa	129	132
AV.IAS675	NA	Avena sativa	Sativa	146	140
AV.IAS676	NA	Avena sativa	Sativa	151	135
AV.IAS677	NA	Avena sativa	Sativa	165	127
AV.IAS678	NA	Avena sativa	Sativa	163	99
AV.IAS679	NA	Avena sativa	Sativa	155	118
AV.IAS680	NA	Avena sativa	Byzantina	145	121
AV.IAS681	NA	Avena sativa	Sativa	150	134
AV.IAS682	NA	Avena sativa	Sativa	164	133
AV.IAS683	NA	Avena sativa	Sativa	171	147
AV.IAS684	NA	Avena sativa	Sativa	138	117
AV.IAS685	NA	Avena sativa	Sativa	159	134
AV.IAS686	NA	Avena sativa	Sativa	161	125
AV.IAS687	NA	Avena sativa	Sativa	156	132
AV.IAS688	NA	Avena sativa	Admixture	133	132
AV.IAS689	NA	Avena sativa	Sativa	164	119
AV.IAS690	NA	Avena sativa	Sativa	160	138
AV.IAS691	NA	Avena sativa	Sativa	143	124
AV.IAS692	NA	Avena sativa	Sativa	144	121
AV.IAS693	NA	Avena sativa	Sativa	146	146
AV.IAS694	NA	Avena sativa	Sativa	143	135
AV.IAS695	NA	Avena sativa	Sativa	139	117
AV.IAS696	NA	Avena sativa	Sativa	162	115
AV.IAS697	NA	Avena sativa	Sativa	156	107
AV.IAS698	NA	Avena sativa	Sativa	161	153
AV.IAS699	NA	Avena sativa	Sativa	146	151
AV.IAS700	NA	Avena sativa	Sativa	149	131
AV.IAS701	NA	Avena sativa	Sativa	140	99
AV.IAS702	NA	Avena sativa	Byzantina	145	128
AV.IAS703	NA	Avena sativa	Sativa	156	131
AV.IAS704	NA	Avena sativa	Admixture	132	126
AV.IAS705	NA	Avena strigosa	Unknown	154	113
AV.IAS706	NA	Avena sativa	Sativa	168	140
AV.IAS707	NA	Avena sativa	Sativa	160	135
AV.IAS708	NA	Avena sativa	Sativa	148	150
AV.IAS709	NA	Avena sativa	Sativa	164	134
